# Supplementary material for: Generation and characterization of iPSC models from HIV-1-positive individuals with divergent clinical outcomes
Source: Stem Cell Reports. 2026 Jan 22;21(2):102786. doi: 10.1016/j.stemcr.2025.102786 (PMC12903088; doi:10.1016/j.stemcr.2025.102786)
Supplement: Document S1. Figures S1–S6, Table S1, and supplemental methods [file mmc1.pdf]

Stem Cell Reports, Volume *21*

Supplemental Information

Generation and characterization of iPSC models from HIV-1-positive individuals with divergent clinical outcomes

Nathalia Almeida, Sam Acors, Daniel Cox, Neophytos Kouphou, Lazaros Fotopoulos, Thomas Williams, Patricia A. Otto, Eun-Young Kim, Steven M. Wolinsky, Davide Danovi, Alessandra Vigilante, Michael H. Malim, and Luis Apolonia

Supplemental Figures

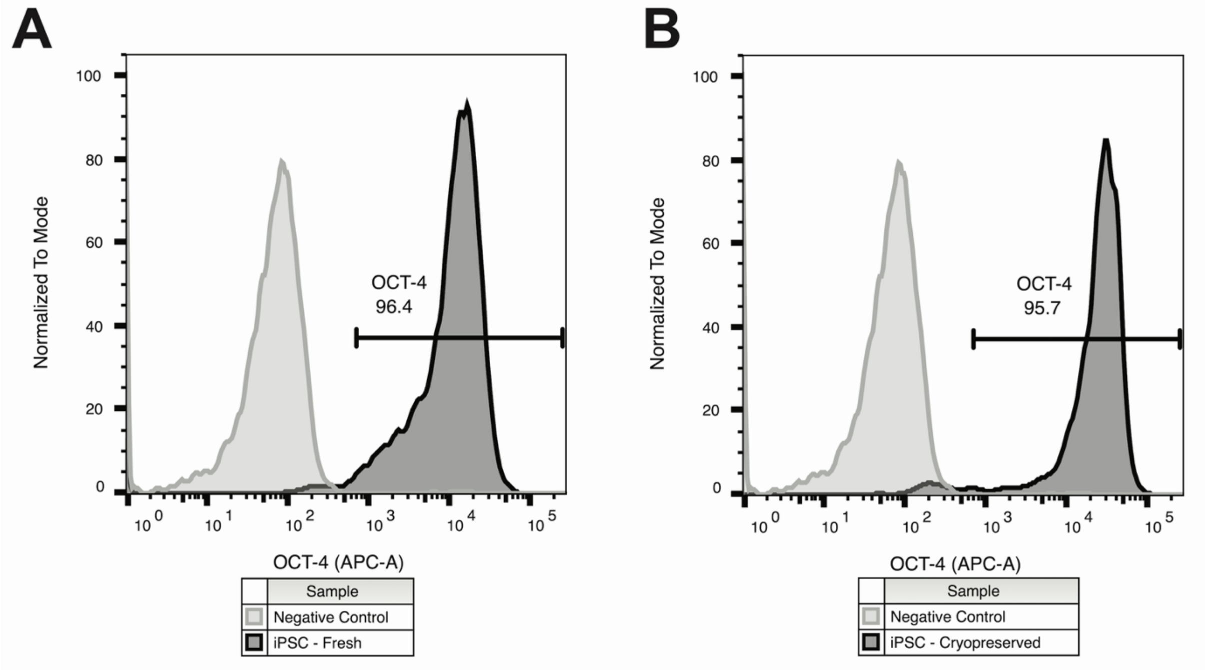

Figure S1. iPSCs generated from fresh or cryopreserved PBMCs express OCT-4. Histogram plots show OCT-4 expression in iPSC lines derived from either (A) fresh or (B) cryopreserved PBMCs, analysed by flow cytometry. iPSC populations (dark grey) are compared to a stained negative control line (HEK293T; light grey), confirming OCT-4 expression in both conditions.

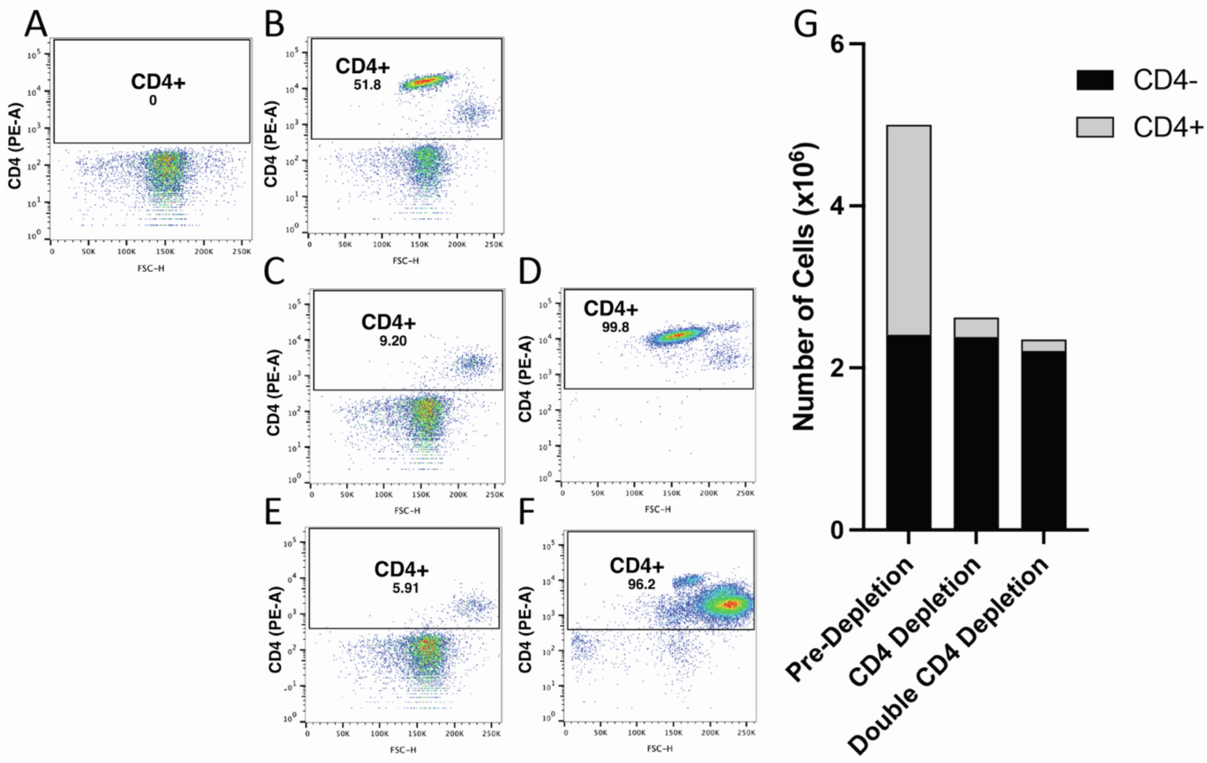

Figure S2. Optimisation of CD4$^+$ cell depletion using magnetic cell sorting.

Flow cytometry plots and a bar graph illustrating the efficiency of CD4$^+$ cell removal from a PBMC sample (healthy donor) following one or two rounds of magnetic depletion. (A) Unstained and (B) CD4-stained PBMCs prior to depletion. (C) CD4$^-$ and (D) CD4$^+$ fractions stained for CD4 after one round of depletion. (E) CD4$^-$ and (F) CD4$^+$ fractions stained after a second depletion round. (G) Total cell counts before depletion, after one round, and after two rounds, with CD4$^+$ cells shown in grey and CD4$^-$ cells in black.

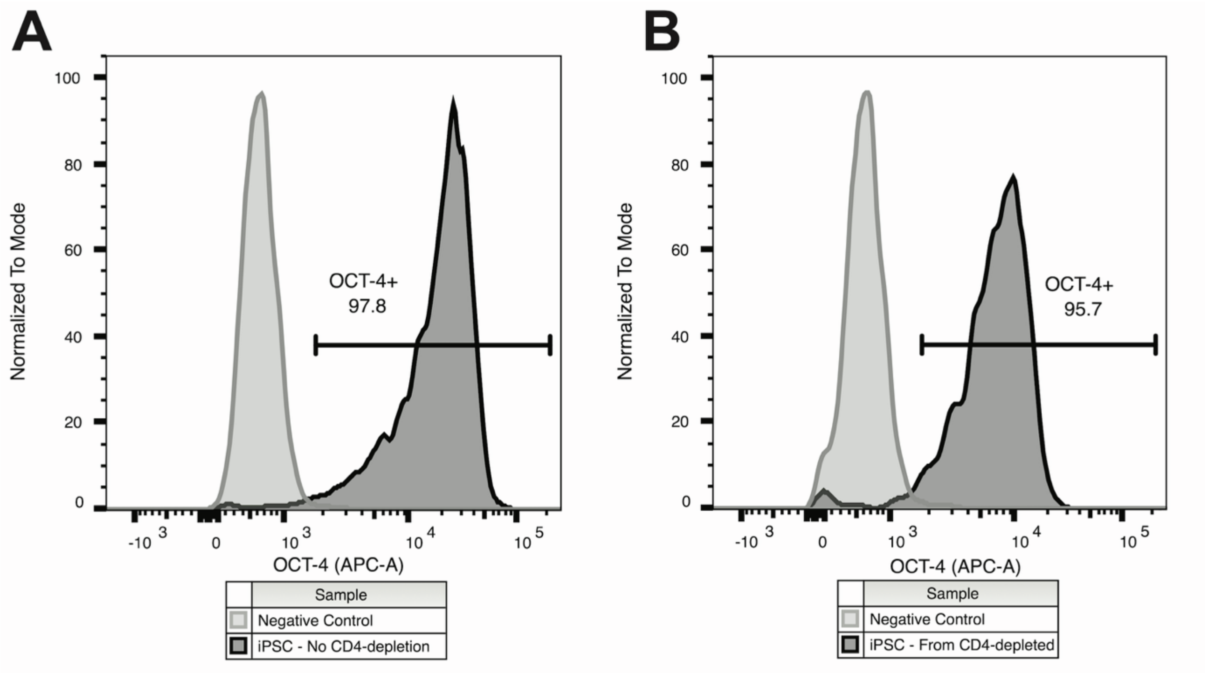

Figure S3. iPSCs generated from whole PBMCs or CD4-depleted PBMCs express OCT-4.

Histogram plots show OCT-4 expression in iPSC lines derived from either (A) whole PBMCs or (B) CD4-depleted PBMCs, analysed by flow cytometry. iPSC populations (dark grey) are compared with a stained negative control line (HEK293T; light grey), confirming robust OCT-4 expression under both reprogramming conditions.

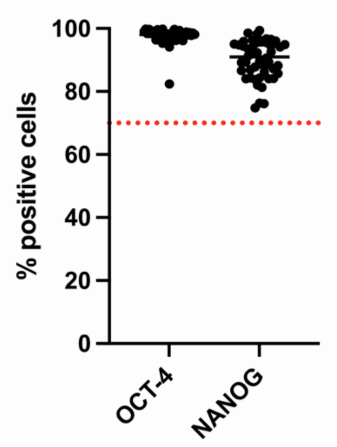

Figure S4. Pluripotency marker expression in MACS-iPSC lines, related to Figure 2.

Quantification of flow cytometry analysis of OCT-4 and NANOG expression across all 50 MACS-iPSC lines generated. The graph shows the percentage of cells positive for each marker, with a red dashed line indicating the 70% threshold required to define pluripotency.

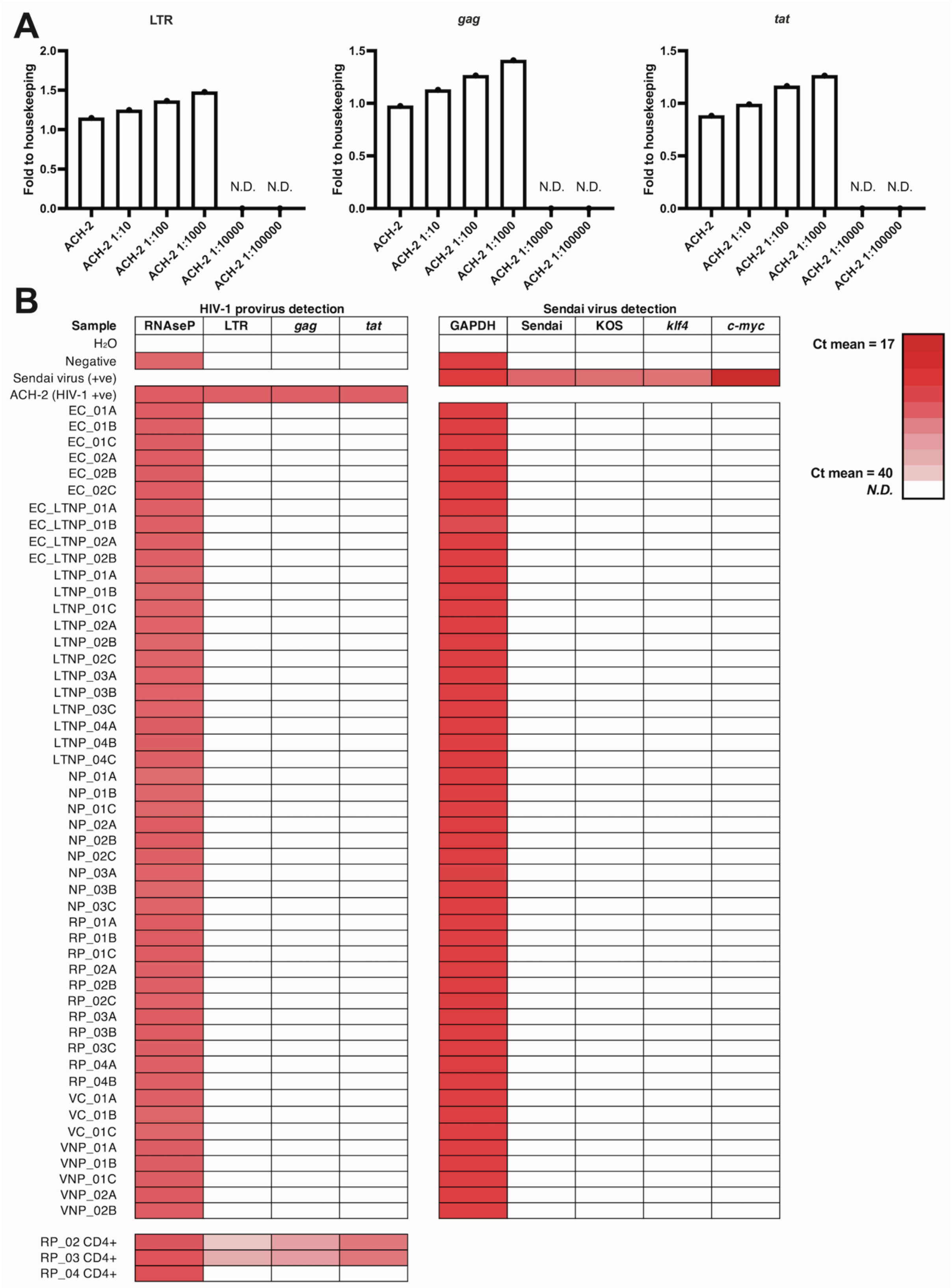

Figure S5. Detection of HIV-1 provirus and Sendai vectors on MACS-iPSC lines, related to Figure 2.

(A) Sensitivity of the HIV-1 proviral DNA qPCR assay. Genomic DNA from ACH-2 cells (one integrated provirus per cell) was serially diluted into HEK293T DNA and analysed by qPCR

targeting conserved regions with the 5' LTR, *gag*, and *tat*. RNaseP served as an internal genomic DNA control. Data are shown as fold change relative to RNaseP. N.D. indicates no detectable amplification. (B) Heatmap summarising qPCR screening of all MACS-iPSC lines for HIV-1 proviral sequences (5' LTR, *gag*, *tat*) and residual Sendai reprogramming vectors (Sendai, KOS, *Klf4*, *c-Myc*). Ct values are shown as a gradient from bright red (Ct = 17) to pale red (Ct = 40); N.D. indicates no detectable amplification. Positive controls included genomic RNA from the Sendai virus vector stock and genomic DNA from ACH-2 cells. Genomic DNA from the CD4+ PBMC fractions obtained during the depletion workflow are shown at the bottom.

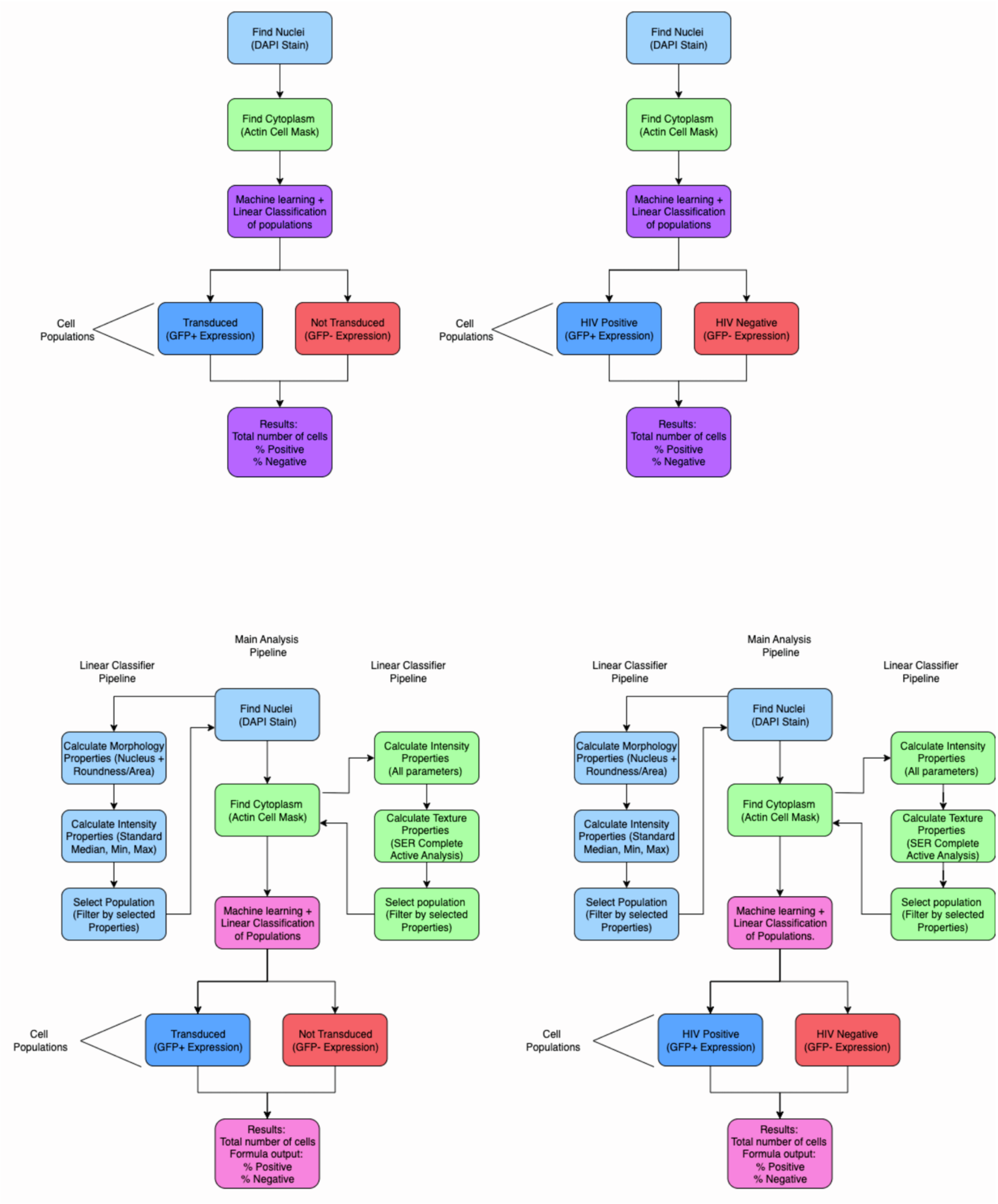

Figure S6. Diagram of Operetta algorithms to quantify transduction/infection. Harmony 4.9 custom pipelines to assess transduction with lentiviral vectors (left) or infection with full-length HIV-1 viruses (right).

Supplemental Tables

Table S1. Primers and probes used for the detection of HIV-1 provirus by qPCR, related to Figures 2 and S5.

Primer name	Primer sequence (5'-3')
HIV-1 LTR forward	CAGATGCTGCATATAAGCAGCTG
HIV-1 LTR reverse	GAGGGATCTCTAGNYACCAGAGT
HIV-1 LTR probe	FAM - CCTGTACTGGGTCTCTCTGG - TAMRA
HIV-1 *gag* forward	ATAATCCACCTATCCCAGTAGGAGAAAT
HIV-1 *gag* reverse	TTTGGTCCTTGTCTTATGTCCAGAATGC
HIV-1 *gag* probe	FAM - ATCCTGGGATTAAATAAAATAGTAAGAATGTATAGCCCTAC - TAMRA
HIV-1 *tat* forward	CCTGTGCCTCTTCAGCTAC
HIV-1 *tat* reverse	ATCCTTTCCAAGCCCTGTCT

Supplemental Methods

HIV-1 molecular clones, lentiviral vectors and production

The pNL4-3 provirus is a full-length, replication-competent HIV-1 molecular clone (Adachi et al., 1986). The CCR5-tropic pNL4-3$_{Bal}$ derivative contains the Bal env (Rasaiyaah et al., 2013). The pYU2 clone derives from the original HIV-1$_{YU2}$ isolate (Fouchier et al., 1997). Lentiviral vector plasmids included p8.91 (pCMV-ΔR8.91), pGFP (pHRsin.SFFV.eGFP), and pVSVg (pMD2.G), as described (Dull et al., 1998; Zufferey et al., 1997).

Lentiviral vectors were generated by co-transfecting HEK293T cells with p8.91, pGFP, and pVSV-G (1:1:0.125) using polyethyleneimine (PolySciences). VSV-G-pseudotyped HIV-1 was produced via co-transfection of pNL4.3 with pVSV-G (20:1). Full-length HIV-1$_{NL43/Bal}$ or HIV-1$_{YU2}$ stocks were produced similarly with the corresponding proviral plasmid. Supernatants were collected 48h post-transfection, filtered (0.45 μm), and concentrated by ultracentrifugation through 20% sucrose (150,000 × g, 75 min, 4 °C). Viral particles were quantified by p24^{Gag} AlphaLISA (PerkinElmer, AL291 C/F) or by titration on HEK293Ts cells.

Three-germ layer differentiation

Three-germ-layer differentiation was performed using the STEMdiff Trilineage Differentiation Kit (STEMCELL Technologies) according to the manufacturer's instructions. iPSCs were seeded onto Matrigel-coated 96-well plates, and, after 24h, cultured in the kit's lineage-specific media. Mesoderm, endoderm, and undifferentiated controls were fixed on day 4; ectoderm cultures were fixed on day 6.

G-banded karyotype analysis

G-banded karyotyping of MACS-iPSC lines (passages 15-30) was performed by Cell Guidance Systems following the provider's instructions.

Quantitative PCR

Genomic DNA and total RNA were extracted using the DNeasy Blood & Tissue Kit (Qiagen) and the Monarch Total RNA Miniprep Kit (New England Biolabs). cDNA was synthesised from 1 μg RNA using the High-Capacity cDNA Reverse Transcription Kit (Thermo Fisher Scientific). HIV-1 provirus was detected by qPCR targeting conserved 5'-LTR and *gag* regions (TaqMan, Thermo Fisher Scientific) and *tat* (PoweUp SYBR Green, Thermo Fisher Scientific) using primers listed in **Table S1**. Sendai virus (SeV) and reprogramming vectors (KOS, KLF4, c-MYC) were detected by TaqMan qPCR on cDNA using pre-designed primers (Thermo Fisher Scientific). Results are reported as mean Ct; samples with no amplification within 40 cycles were recorded as not determined (N.D.).

Flow cytometry

iPSCs were dissociated with GCDR, fixed, permeabilised with 0.1% Triton X-100 (Sigma-Aldrich), and blocked in 2% bovine serum albumin (BSA, Sigma-Aldrich). Cells were stained with PE-conjugated anti OCT-4 (1:100, BioLegend, 653704) or NANOG (1:100, Invitrogen, PA5-46891) in 2% BSA/PBS for 30 min at RT in the dark, washed in MACS buffer (PBS + 0.5% BSA + 2 mM EDTA, Gibco), and analysed by flow cytometry. MACS-macrophages (7 days) were detached with 6 mg/mL lidocaine (Sigma-Aldrich) and 10 mM EDTA, incubated with Zombie NIR live/dead (Biolegend) for 15 min, blocked with FcR reagent (Miltenyi Biotec) for 20 min at 4°C, and stained with antibodies in MACS buffer for 30 min at 4°C: CD14-BV711 (Biolegend, 301838), CD16-PE/Cy7 (Biolegend, 302016), CD206-APC/Cy7 (Biolegend, 321120), CD163-APC (Biolegend, 333610), CD68-PE/Cy7 (Biolegend, 333816), CD4-PE (Biolegend, 300508), CCR5-APC (Biolegend, 359122), CXCR4-PE/Cy7 (Biolegend, 306514). All samples were analysed on a CytoFLEX Flow Cytometer (Agilent) and processed using FlowJo v10.

Immunofluorescence

Fixed cells were permeabilised and blocked in 1x Perm/Wash buffer (Biolegend) plus 10% FBS. Primary antibodies were incubated overnight at 4°C: OCT-4 (rabbit 1:500, Abcam, ab19857), PAX-6 (mouse 1:200, Abcam, ab78545), BRACHYURY (goat 1:40, Bio-Techne, AF2085), and SOX-17 (goat 1:250, Bio-Techne, AF1924), GFP (rabbit 1:100, Thermo Fisher Scientific, G10362), or PE-p24Gag (1:200, KC57-RD1, Beckman Coulter, 6604667). Appropriate Alexa Fluor-conjugated secondary antibodies were used at 1:500, and nuclei and cytoplasm were counterstained with DAPI and CellMask Deep Red (Invitrogen, A57258). Images were acquired on an Operetta CLS (20x) and analysed using Harmony 4.9 custom pipelines detecting nuclei, cytoplasm, and GFP or Gag per cell (see **Figure S6**), or on a Leica TCS SP8 confocal microscope.

Phagocytosis assay

MACS-macrophages and MDMs (7 days) or undifferentiated iPSCs were incubated with Alexa Fluor 488-conjugated Zymosan A particles (Invitrogen, Z23373) at a 2:1 particle-to-cell ratio for 30 min at 37°C. Non-internalised particles were quenched with 250 μg/mL trypan blue/PBS for 5 min, followed by a PBS wash. As a negative control, MACS-macrophages were pre-treated with 10 μM Cytochalasin D (Tocris, 1233) for 1 h. Cells were analysed by flow cytometry (CytoFLEX, Agilent).

Supplemental References

Adachi, A., Gendelman, H.E., Koenig, S., Folks, T., Willey, R., Rabson, A., and Martin, M.A. (1986). Production of acquired immunodeficiency syndrome-associated retrovirus in human and nonhuman cells transfected with an infectious molecular clone. J Virol *59*, 284–291. https://doi.org/10.1128/jvi.59.2.284-291.1986.

Dull, T., Zufferey, R., Kelly, M., Mandel, R.J., Nguyen, M., Trono, D., and Naldini, L. (1998). A third-generation lentivirus vector with a conditional packaging system. J Virol *72*, 8463–8471. https://doi.org/10.1128/JVI.72.11.8463-8471.1998.

Fouchier, R.A., Meyer, B.E., Simon, J.H., Fischer, U., and Malim, M.H. (1997). HIV-1 infection of non-dividing cells: evidence that the amino-terminal basic region of the viral matrix protein is important for Gag processing but not for post-entry nuclear import. EMBO J *16*, 4531–4539. https://doi.org/10.1093/emboj/16.15.4531.

Rasaiyaah, J., Tan, C.P., Fletcher, A.J., Price, A.J., Blondeau, C., Hilditch, L., Jacques, D.A., Selwood, D.L., James, L.C., Noursadeghi, M., et al. (2013). HIV-1 evades innate immune recognition through specific cofactor recruitment. Nature *503*, 402–405. https://doi.org/10.1038/nature12769.

Zufferey, R., Nagy, D., Mandel, R.J., Naldini, L., and Trono, D. (1997). Multiply attenuated lentiviral vector achieves efficient gene delivery in vivo. Nat Biotechnol *15*, 871–875. https://doi.org/10.1038/nbt0997-871.
