# Supplementary material for: Generation and characterization of iPSC models from HIV-1-positive individuals with divergent clinical outcomes
Source: Stem Cell Reports. 2026 Jan 22;21(2):102786. doi: 10.1016/j.stemcr.2025.102786 (PMC12903088; doi:10.1016/j.stemcr.2025.102786)
Supplement: Document S2. Article plus supplemental information [file mmc2.pdf]

STEM CELL REPORTS

Resource

OPEN ACCESS

Generation and characterization of iPSC models from HIV-1-positive individuals with divergent clinical outcomes

Nathalia Almeida,[1,4] Sam Acors,[1,4] Daniel Cox,[1] Neophytos Kouphou,[1] Lazaros Fotopoulos,[2] Thomas Williams,[2] Patricia A. Otto,[3] Eun-Young Kim,[3] Steven M. Wolinsky,[3] Davide Danovi,[2] Alessandra Vigilante,[2] Michael H. Malim,[1,*] and Luis Apolonia[1,2,5,*]

[1]Department of Infectious Diseases, School of Immunology and Microbial Sciences, King's College London, London SE1 9RT, UK
[2]Centre for Gene Therapy & Regenerative Medicine, School of Basic & Medical Biosciences, King's College London, London SE1 9RT, UK
[3]Department of Medicine, Northwestern University Feinberg School of Medicine, Chicago, IL 60611, USA
[4]These authors contributed equally
[5]Lead contact
*Correspondence: michael.malim@kcl.ac.uk (M.H.M.), luis.apolonia@kcl.ac.uk (L.A.)
https://doi.org/10.1016/j.stemcr.2025.102786

SUMMARY

The clinical outcome of human immunodeficiency virus type-1 (HIV-1) infection varies greatly among individuals, ranging from rapid disease progression to natural viral suppression. While viral and environmental factors contribute, host genetics are considered major determinants of disease trajectory. To enable mechanistic studies of host factors underlying disease outcomes, we generated 50 induced pluripotent stem cell (iPSC) lines from 18 participants of the Multicenter AIDS Cohort Study (MACS), spanning a spectrum of clinical trajectories. Reprogrammed MACS lines are confirmed to be HIV-1 negative and Sendai vector-free. We validate their pluripotency and demonstrate robust differentiation into macrophages capable of productive HIV-1 infection. These MACS-iPSC lines offer a genetically diverse resource to model HIV-1 infection *in vitro*, where clinical progression is known. Crucially, their capacity to differentiate into HIV-1 target cells and other disease-relevant lineages makes them a powerful tool to uncover host determinants of HIV-1 pathogenesis and advance targeted treatment and curative strategies.

INTRODUCTION

Despite decades of extensive research and the effectiveness of anti-retroviral therapy (ART), human immunodeficiency virus type-1 (HIV-1) infection remains a major global health challenge (UNAIDS). When left untreated, HIV-1 infection almost always leads to acquired immunodeficiency syndrome (AIDS), defined by a plasma CD4$^+$ T cell count below 200 cells/μL, resulting in susceptibility to fatal opportunistic infections (Boshoff and Weiss, 2002; Picker, 2006). A hallmark of HIV-1 pathogenesis is the wide variability in disease progression (Sabin and Lundgren, 2013; Touloumi et al., 2013). A small subset (<1%), known as elite controllers (ECs), maintain undetectable levels of plasma HIV-1 RNA (<50 copies/mL) without ART. In contrast, rapid progressors (RPs) control viral replication poorly and experience rapid immunological decline, developing AIDS within 2 or 3 years of seroconversion and/or exhibiting a steep CD4$^+$ T cell loss early in infection (Eisinger et al., 2019; Quinn et al., 2000). Understanding the underlying factors contributing to this spectrum of clinical outcomes remains a central goal in AIDS research.

Prospective longitudinal HIV-1 cohorts such as the Multicenter AIDS Cohort Study (MACS), the Women's Interagency HIV Study (WIHS), and the Swiss HIV Cohort Study (SHCS) have been instrumental in advancing our understanding of HIV-1 disease and uncovering host and viral determinants that influence disease progression (Jiang et al., 2013; Kulkarni et al., 2019; McLaren and Carrington, 2015). Specifically, the MACS, established in 1984 in the United States and comprising the longest running observational HIV-1 study, stands out for its unparalleled longitudinal depth. The study follows men at high risk of HIV-1 infection, tracking both natural and treated disease courses (Kaslow et al., 1987). Over decades, the study has accumulated detailed clinical, immunological, virological, and behavioral data, alongside cryopreserved peripheral blood mononuclear cells (PBMCs) (Barkan et al., 1998; D'Souza et al., 2021; Egger et al., 1997; Kaslow et al., 1987). Clinical records capture AIDS-defining events and ART initiation, while molecular data include CD4$^+$ T cell counts and viral load. This combination makes MACS a unique resource for studying diverse clinical trajectories of HIV-1 infection.

In addition to the extremes of RPs and ECs, the MACS study also classified individuals who remained AIDS-free without ART as non-progressors (NPs), and those who maintained this status for over 15 years were termed long-term non-progressors (LTNPs). Viremic controllers (VCs) were defined by consistently low but detectable viral loads (<2,000 HIV-1 RNA copies/mL), while a subset of VCs, termed viremic non-progressors (VNPs), maintained high levels of viremia (>10,000 HIV-1 RNA copies/mL) without AIDS-defining illnesses and stable CD4$^+$ T cell counts for over 8 years, resembling the non-pathogenic simian immunodeficiency virus infection of sooty mangabeys (SIV_{SM})

© 2026 The Authors.
Published by Elsevier Inc. on behalf of International Society for Stem Cell Research.
This is an open access article under the CC BY license (http://creativecommons.org/licenses/by/4.0/).

(Silvestri et al., 2003). Of note, these classifications can overlap; for example, some ECs are also LTNPs.

Both viral and host genetics influence HIV-1 disease progression. Population studies estimate that the viral genotype explains only ~30%–50% of the variance in set-point viral load (Alizon et al., 2010; Blanquart et al., 2017; Fraser et al., 2014), implying a substantial contribution of host and environmental factors. Genome-wide association studies (GWAS) have identified several loci associated with viral control, most notably within the human leukocyte antigen (HLA) region (McLaren and Fellay, 2021). Alleles such as *HLA-B*57*:01, *B*27*, and certain *HLA-C* variants correlate with slower disease progression, while *HLA-B35* associates with rapid progression (Kaslow et al., 1996). The *CCR5Δ32* deletion, affecting the major HIV-1 entry co-receptor, confers resistance to HIV-1 (Dean et al., 1996; Huang et al., 1996; Liu et al., 1996). However, these variants explain only a fraction of outcome variability (An and Winkler, 2010; The International HIV Controllers Study, 2010), suggesting additional host mechanisms.

Linking clinical phenotypes to immune cell behaviors *in vitro* is challenging because viral replication varies across primary cultures, even within the same donor, and primary cells have limited lifespan. Induced pluripotent stem cells (iPSCs) overcome these limitations: PBMCs can be reprogrammed to a pluripotent state by ectopic expression of OCT-4, SOX2, KLF4, and c-MYC (Takahashi et al., 2007; Yu et al., 2007). iPSCs retain the donor's genetic background, expand indefinitely, and differentiate into relevant immune lineages, offering a consistent platform for probing intrinsic cellular susceptibilities and responses to HIV-1.

Given their broad potential, iPSCs can be used across a wide range of fields, from regenerative medicine to disease modeling and drug discovery (Shi et al., 2017). Previous studies have generated iPSCs from HIV-1-infected patients to explore HIV-1 pathogenesis, including *CCR5*-deficient iPSC-derived macrophages resistant to HIV-1 and brain organoid models of HIV-1 neuropathogenesis (Kambal et al., 2011; Teque et al., 2020; Wei et al., 2023; Ye et al., 2014). However, their use in uncovering the molecular interactions between HIV-1 and its host, and variations thereof, remains largely underexplored.

Here, we describe iPSC lines generated from PBMCs of MACS participants with well-defined, extreme HIV-1 clinical outcomes. This panel provides a model for investigating the cellular and genetic basis of inter-individual differences in HIV-1 replication and disease progression. Although this resource could have been used to derive iPSCs harboring proviruses for studying latency and reactivation, our work was designed to address a distinct biological question. In particular, because we wish to study host-intrinsic control of HIV-1 replication, the establishment of HIV-1-free lines was essential. This panel of iPSC lines offers an experimental platform to investigate genetic and cellular mechanisms linking host biology to HIV-1 disease outcomes.

RESULTS

Selection of MACS study participants for iPSC generation

To generate a panel of iPSC lines from the well-characterized cohort of individuals with diverse HIV-1 disease trajectories, we explored the depth of longitudinal data available from the MACS, focusing on participants with extreme clinical phenotypes. Such individuals offer the greatest potential for identifying host factors and cellular mechanisms that underpin these distinct disease outcomes. We selected 18 donors spanning the spectrum from ECs to RPs for PBMC reprogramming. To illustrate the differences between these extremes, we plotted $CD4^+$ T cell counts and viral RNA levels. Figure 1A shows the steep decline in $CD4^+$ T cell count in a representative RP over the years and the corresponding overall increase in viral load. In contrast, a typical EC maintained a stable $CD4^+$ T cell count over a similar time frame in the absence of ART, with undetectable viremia (Figure 1B). Table 1 summarizes donor characteristics, PBMC sample details, HIV-1 status, and ART use. Among the 18 individuals, we selected 4 RPs, 4 ECs (2 of whom were also LTNPs), 3 NPs, 4 LTNPs, 1 VC, and 2 VNPs. PBMCs were collected between 1985 and 2014, with 14/18 samples obtained after seroconversion.

Prior to reprogramming the selected PBMC samples, we conducted preliminary experiments to address potential challenges associated with using long-term cryopreserved and HIV-1-positive specimens. Our method of choice was reprogramming via transduction with an engineered Sendai virus cocktail encoding *OCT-4*, *SOX2*, *KLF4*, and *c-MYC*, a non-integrating and highly efficient method chosen for its reproducibility with available commercial kits, and feeder-free culture to minimize xenogeneic effects, enhance cell purity, and avoid culturing mouse embryonic fibroblasts (Ban et al., 2011; Chen et al., 2011; Fusaki et al., 2009). Median cell recovery was 81.1%, and the median viability was 94%, with consistent viability regardless of storage duration, HIV-1 status, or $CD4^+$ T cell count. We examined the impact of cryopreservation on iPSC generation and found no differences in efficiency between fresh and cryopreserved PBMCs from a healthy donor (Figure S1).

Since most samples were collected after HIV-1 diagnosis, we sought to minimize the risk of generating iPSCs harboring HIV-1 provirus; this was important as our objective was to generate HIV-1-free iPSC lines suitable for

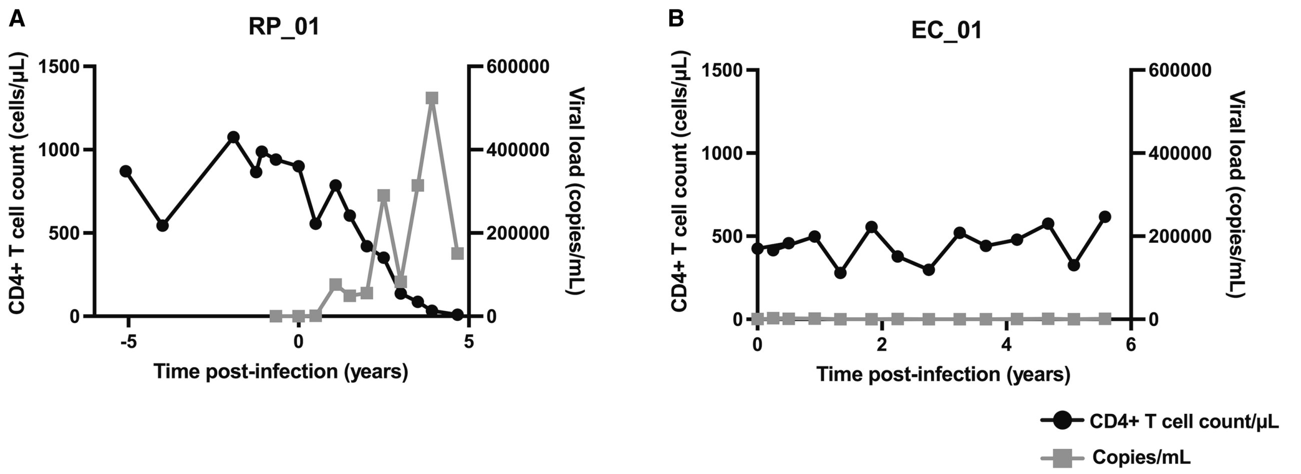

Figure 1. Longitudinal data of two MACS participants show opposing clinical outcomes to HIV-1 infection
(A) CD4$^+$ T cell counts (black circles, left *y* axis, cells/µL) and plasma viral load (gray squares, right *y* axis, copies/mL) over time post-infection for donor RP_01 (rapid progressor).
(B) CD4$^+$ T cell counts (black circles, left y axis, cells/µL) and plasma viral load (gray squares, right *y* axis, copies/mL) over time post-infection for donor EC_01 (elite controller).

studying host susceptibility in relation to clinical outcomes. Because HIV-1 infects CD4$^+$ cells, we optimized a CD4$^+$ cell depletion protocol, finding that two rounds of magnetic cell sorting were necessary. The first round removed most CD4$^+$ T cells (>99%) and some monocytes, while the second round further depleted monocytes (from 9.2% to 5.91% of total CD4$^+$ cells) and residual CD4$^+$ T cells to undetectable levels (Figure S2). Reprogramming of PBMCs from healthy donors with or without CD4$^+$ depletion yielded iPSCs with comparable OCT-4 expression (Figure S3), validating the use of depletion to minimize potential proviral carryover.

Generation of iPSCs from CD4-depleted PBMCs of MACS participants

After optimizing the protocol, we thawed the selected MACS samples, double-depleted CD4$^+$ cells, and transduced them with the recombinant Sendai virus cocktail. All 18 donor PBMCs were successfully reprogrammed into iPSCs (MACS-iPSCs). We selected at least two clones per cell line: 14 donors yielded three clones, and four donors (RP_04, EC_LNTP_01, EC_LNTP_02, and VNP_02) yielded two clones, resulting in a panel of 50 MACS-iPSC lines (designated "MACS ID" followed by A, B, or C).

To confirm the authenticity of the iPSC lines, we performed several quality control steps. First, we assessed colony morphology using light microscopy, confirming the expected round, compact colony morphology with smooth, defined edges and no signs of overgrowth or differentiation. A representative bright-field image of line RP_02A is shown in Figure 2A, which will be used as a reference hereafter. Second, we assessed pluripotency marker expression by quantifying OCT-4 and NANOG expression using flow cytometry. All lines showed higher than 70% expression for both markers (Figures 2B and S4), exceeding the threshold for pluripotency (Sullivan et al., 2018).

Reprogramming can result in genetic abnormalities, including cancer-driving mutations, independent of the methodology used (Avior et al., 2021; D'Antonio et al., 2018; Wang et al., 2017). To evaluate the chromosomal integrity of the MACS-iPSCs, we performed karyotyping in a total of 20 cells from each line. A representative karyotype from RP_02A is shown in Figure 2C, displaying the stable 46, XY chromosome integrity, also observed in 41/50 lines. Eight lines displayed common iPSC-associated abnormalities, while EC_LNTP_02B showed a rare mosaic gain of chromosome 8 in 2/20 cells (Table 2).

Finally, we assessed MACS-iPSC lines for residual Sendai virus and HIV-1 provirus to ensure these would not interfere with downstream virus infection assays. We evaluated conserved HIV-1 long terminal repeat (LTR), *gag*, and *tat* sequences using validated primers (Ou et al., 1988; Ye et al., 2020), first establishing assay sensitivity with serial dilutions of ACH-2 cells, which contain one integrated HIV-1 provirus per cell. Our assay could readily detect HIV-1 provirus-containing cells in a background of 1,000 provirus-null cells (Figure S5A). All MACS-iPSC lines were negative for both HIV-1 provirus and Sendai virus (Figures 2D and S5B). Positive controls also included DNA from the CD4$^+$ PBMC fraction of three donors, including one sample collected prior to HIV-1 infection as a negative control. HIV-1 provirus was detected only in the expected positive

Table 1. Details of MACS PBMC samples selected to be reprogrammed into iPSCs

MACS donor	Sample collection date	Post HIV-1 infection?	ART status at sampling
EC_01	18/02/2014	yes	no
EC_02	03/09/2003	yes	yes
EC_LNTP_01	16/04/2003	yes	no
EC_LNTP_02	10/04/2012	yes	no
LNTP_01	25/04/1989	yes	no
LNTP_02	18/07/2001	yes	no
LNTP_03	12/09/2006	yes	no
LNTP_04	01/05/2007	yes	yes
NP_01	10/06/1986	no	N/A
NP_02	09/02/1993	no	N/A
NP_03	19/11/1998	yes	yes
RP_01	02/07/1990	yes	no
RP_02	22/02/1999	yes	yes
RP_03	12/04/1999	yes	yes
RP_04	02/04/1985	no	N/A
VC_01	21/01/1988	no	N/A
VNP_01	03/07/1986	yes	no
VNP_02	05/06/1992	yes	no

RP, rapid progressor; EC, elite controller; LTNP, long-term non-progressor; VC, viremic controller; VNP, viremic non-progressor.

controls, and its absence from the pre-infection sample confirms the assay's specificity.

Taken together, these findings confirm the successful generation of 50 new iPSC lines from 18 MACS cohort participants.

Pluripotency validation of MACS-iPSCs and differentiation into specialized cell types

iPSCs are powerful tools for *in vitro* disease modeling due to their capacity to differentiate into a wide range of cell types. To confirm that our newly reprogrammed MACS-iPSC lines retain this ability, we confirmed their pluripotency with a commercially available three-germ layer differentiation assay. Successful differentiation was determined by lineage-specific marker expression via immunofluorescent staining, with undifferentiated iPSCs serving as negative controls.

In the undifferentiated state, iPSCs showed the expected high expression of the pluripotency marker OCT-4, as shown in Figure 2B and further confirmed in Figure 3A. To direct their identity toward the ectoderm, mesoderm, or endoderm lineages, we cultured these cells with lineage-specific differentiation media containing defined cytokines and subsequently imaged for lineage markers. Confocal microscopy of grayscale maximum-intensity z stack projections confirms loss of OCT-4 expression, indicating exit from the pluripotent state (Figure 3A). Differentiated cells exhibited robust expression of lineage-specific markers, namely PAX-6 (ectoderm), BRACHYURY (mesoderm), and SOX-17 (endoderm), as shown in Figures 3B–3D. These findings establish that these MACS-iPSCs possess full pluripotency capacity and can give rise to all three germ layers, validating their potential for *in vitro* differentiation into specialized cell types.

Having confirmed the pluripotency of MACS-iPSCs, we next sought to demonstrate their capacity to differentiate into a specialized cell type that can naturally support HIV-1 infection and replication. Although $CD4^{+}$ T cells are the primary *in vivo* targets, their generation from iPSCs remains technically challenging. Existing differentiation protocols are labor intensive, and only recently, feeder- and serum-free methods have been developed—approaches that are still being optimized for yield, consistency, and clinical applicability (Fong et al., 2025). By contrast, macrophages, key innate immune cells in HIV-1 pathogenesis, can be reliably derived from iPSCs. First described over a decade ago (Wilgenburg et al., 2013), iPSC-derived macrophages are well characterized and demonstrate significant promise in clinical applications, from drug screening to disease modeling (Gutbier et al., 2020; Haake et al., 2020; Zhang et al., 2015). We therefore explored the derivation of macrophages from our MACS-iPSC lines.

For proof of principle, we selected four lines from individuals with contrasting clinical outcomes, two ECs and two RPs, and differentiated them into macrophages using a feeder-free protocol adapted from Wilgenburg et al. (2013), with minor modifications to starting cell number for embryoid body formation. Differentiated cells displayed characteristic macrophage morphology (Figure 4A, representative from two MACS-iPSC lines), consisting of an elongated cytoplasm with visible vacuoles (Wilgenburg et al., 2013). Flow cytometry confirmed expression of canonical myeloid/macrophage markers CD14, CD16, CD206, CD163, and CD68 (Figure 4B), consistent with previous reports. Marker expression levels were comparable to those observed in peripheral blood monocyte-derived macrophages (MDMs) and absent from undifferentiated MACS-iPSCs, confirming a successful transition from a pluripotent to a terminally differentiated state.

To validate macrophage identity, we assessed the phagocytic capacity of MACS-iPSC-derived macrophages, a

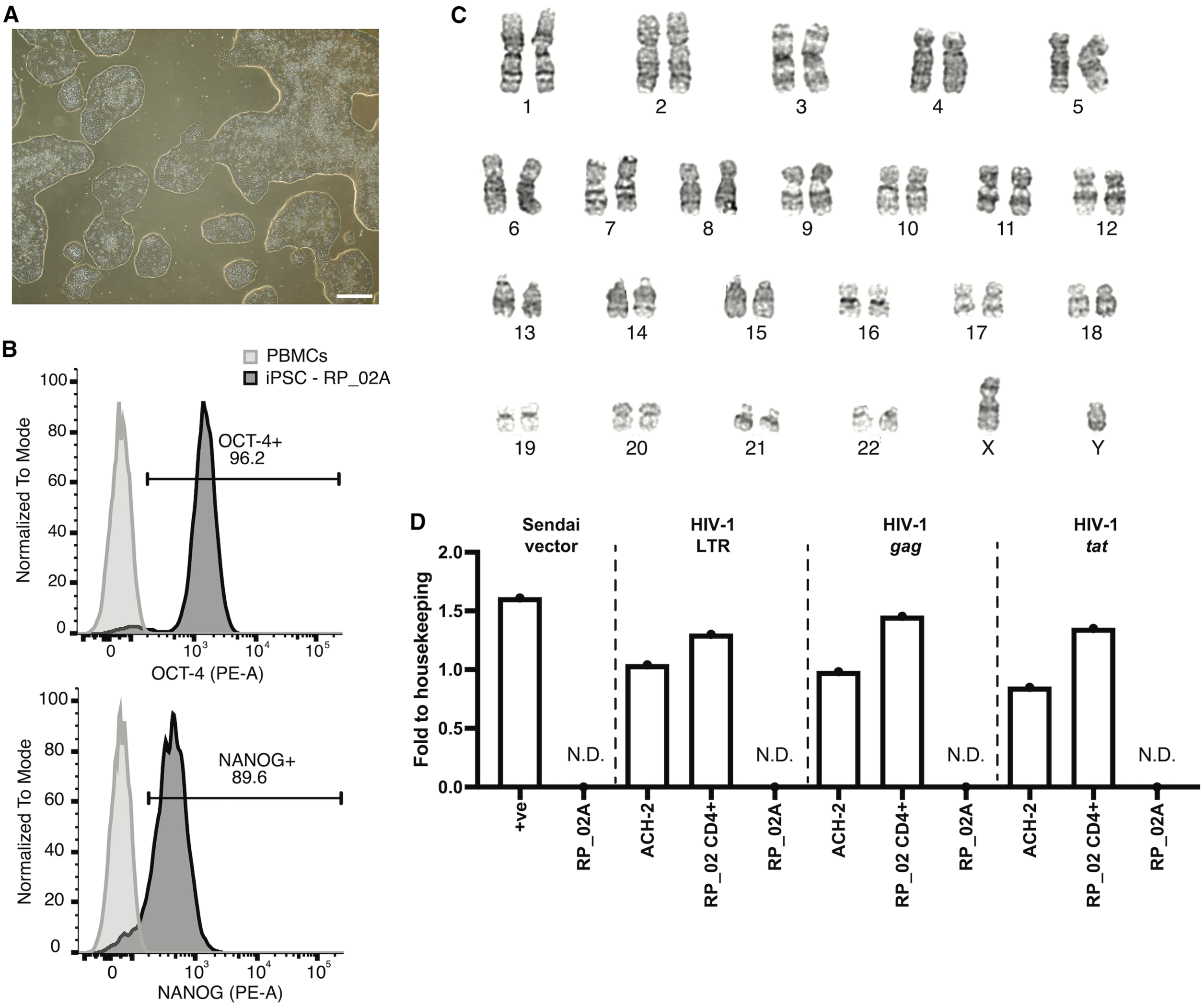

Figure 2. Representative MACS-iPSC line shows successful reprogramming from long-term cryopreserved HIV-1-positive PBMCs using Sendai vectors in a feeder-free protocol

(A) Representative light microscopy image of MACS iPSC line RP_02A. Scale bars, 500 μm.

(B) Flow cytometry histogram showing mean fluorescent intensity of OCT-4 (top) and NANOG (bottom) in MACS-iPSC line RP_02A. Light gray, negative control (PBMCs); dark gray, RP_02A.

(C) G-banded karyotyping analysis of MACS-iPSC line RP_02A.

(D) qPCR detection of Sendai vector and HIV-1 provirus in the MACS-iPSC RP_02A line, shown as fold change relative to housekeeping genes (GAPDH for Sendai; RNaseP for HIV-1). +ve, Sendai virus-positive control. HIV-1 provirus was assessed using primers for LTR, *gag*, and *tat*; ACH-2 and $CD4^+$ MACS-PBMC fraction from donor RP_02 serve as positive controls; $n = 3$ technical replicates, N.D., not detected.

hallmark function of this cell type. After incubation with yeast-derived Alexa Fluor 488-labeled zymosan A particles, we measured uptake by flow cytometry, using MDMs and undifferentiated MACS-iPSCs as positive and negative controls, respectively (Figure 4C). MACS-derived macrophages demonstrated robust phagocytic activity, comparable to that of MDMs, with zymosan A uptake ranging from 40% to 65% across the four different lines. This activity was significantly reduced when cells were treated with cytochalasin D, an actin polymerization inhibitor, or incubated at 4°C, which inhibits energy-dependent phagosome formation, thereby confirming that uptake was both active and cytoskeleton dependent. In contrast, undifferentiated MACS-iPSCs did not exhibit zymosan A uptake, consistent with their non-phagocytic ability.

In summary, these findings corroborate the successful generation of a specialized and functional cell type from MACS-iPSCs and demonstrate the reproducibility of the macrophage differentiation protocol across distinct donor backgrounds. As macrophages are natural targets of HIV-1

Table 2. Karyotype results from MACS-iPSCs showing abnormal karyotypes (20 cells total analyzed per donor)

Cell line	Mutation	Sample date	Phenotype	Reported in stem cells?
NP_01B	46,X,der(Y)t(Y;1)(q12;q12)[20]	1986	NP	yes
VC_01C	47,XY,+12[3]/46,XY[17]	1988	VC	yes
LNTP_02C	46,X,der(Y)t(Y;1)(q12;q12)[3]/46,XY[17]	2001	LTNP	yes
RP_03C	45,X,-Y,i(20)(q10)[2]/46,XY,i(20)(q10)[1]/46,XY[17]	1999	RP	yes
LNTP_03C	46,XY,der(1;10)(q10;q10)[20]	2006	LTNP	yes
NP_03B	47,XY,+i(1)(q10)[1]/46,XY[19]	1998	NP	yes
NP_03C	46,XY,del(18)(q21.3)[20]	1998	NP	yes
LNTP_04C	47,XY,+12[13]/46,XY[7]	2007	LTNP	yes
EC_LNTP_02B	47,XY,+8[2]/46,XY[18]	2012	EC + LTNP	no

The number in square brackets indicates the number of cells exhibiting the described karyotype.

infection, capable of supporting viral replication, MACS-iPSC-derived macrophages offer a biologically relevant *in vitro* model for studying host-virus interactions and disease mechanisms.

Use of MACS-iPSC lines as a research tool for HIV-1 studies

To validate the application of MACS-iPSC lines for studying HIV-1 infection, we first demonstrated that all four MACS-iPSC lines described in the previous section can be transduced as both undifferentiated iPSCs or macrophages using a VSV-G-pseudotyped HIV-1-based lentiviral vector encoding green fluorescent protein (GFP) (Figures 5A and 5B). This highlights the flexibility of the system for genetic manipulation, such as stable gene overexpression using lentiviral vectors. We next evaluated the ability of MACS-iPSCs and MACS-derived macrophages to support infection with replication-competent HIV-1. Flow cytometry analysis confirmed that iPSCs lacked surface expression of the canonical HIV-1 entry receptor CD4 and co-receptor CCR5, while showing low levels of CXCR4 (Figure 5C), confirming that these cells are not naturally permissive to infection with wild-type HIV-1 strains. Nonetheless, infection was achievable using VSV-G-pseudotyped HIV-1_{NL4-3}, with intracellular Gag detectable 48 h post-infection by indirect immunofluorescence (Figure 5D).

In contrast, MACS-derived macrophages expressed CD4, CCR5, and CXCR4 at levels comparable to MDMs (Figure 5C), confirming the presence of HIV-1 entry receptors. To examine whether MACS-derived macrophages support productive HIV-1 infection, we challenged cells with two macrophage-tropic, replication-competent HIV-1 strains (HIV-$1_{NL4-3/BaL}$ and HIV-1_{YU2}) and then quantified intracellular Gag over 6 days post-infection by immunofluorescence. All four MACS-iPSC-derived macrophage lines were successfully infected by both strains, with the percentage of Gag-positive cells steadily increasing, indicating spreading infection and active viral replication of both strains (Figures 5E and 5F). These findings demonstrate that MACS-iPSC-derived macrophages support HIV-1 infection and replication, validating their utility for downstream mechanistic studies. Taken together, our findings outline how this system offers a relevant *in vitro* platform for studying HIV-1 molecular pathogenesis and opens avenues for investigating the molecular and cellular basis of variation in the clinical trajectory of patients infected with HIV-1.

DISCUSSION

Our study describes the generation of a panel of 50 iPSC lines from individuals with distinct HIV-1 disease trajectories, providing a genetically diverse and clinically relevant platform to study HIV-1 pathogenesis. These lines enable investigation of host-intrinsic factors influencing susceptibility and replication, free from donor-specific proviral genomes. While iPSC models retaining integrated proviruses provide valuable tools for investigating HIV-1 latency and reactivation dynamics, our provirus-free approach complements these efforts, focusing on host determinants of infection. We anticipate that the MACS-iPSC resource will facilitate linking cellular phenotypes to clinical outcomes, enabling the identification of novel host determinants relevant to understanding variation in HIV-1 transmission and disease course.

All participants were selected from the MACS cohort, which provides extensive clinical data and cryopreserved

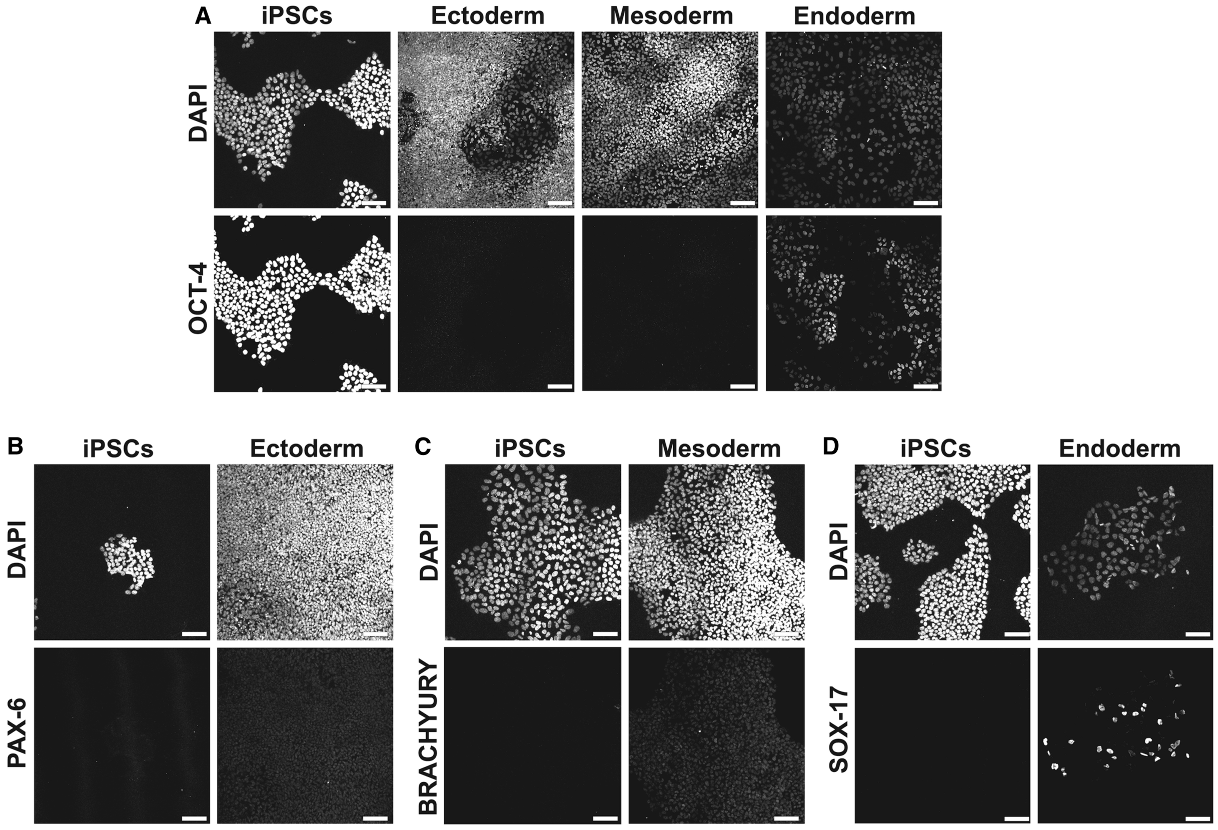

Figure 3. Three-germ layer differentiation assay confirms pluripotency of MACS-iPSC lines

Representative confocal images of MACS-iPSC line RP_02A (grayscale, maximum-intensity z stack projection) showing.

(A) DAPI (top) and OCT-4 (bottom) in undifferentiated iPSCs and after differentiation.

(B) DAPI and PAX-6 (ectoderm marker).

(C) DAPI and BRACHYURY (mesoderm marker).

(D) DAPI and SOX-17 (endoderm marker).

Scale bars, 100 μm.

PBMCs from natural and treated HIV-1 histories. To overcome limitations in PBMC-based studies, including the finite number of experiments that can be performed with a single blood draw, and extend the utility of these irreplaceable samples, we reprogrammed PBMCs from selected participants into iPSCs, generating a scalable and highly versatile resource. iPSCs offer the unique advantage of self-renewal and pluripotency, enabling the derivation of a broad range of cell types.

We established a robust and optimized feeder-free pipeline for reprogramming long-term cryopreserved, HIV-1-positive PBMC samples into Sendai-free, HIV-1 provirus-negative iPSCs. Although the precise cell type(s) within a heterogeneous PBMC fraction that give rise to iPSCs is unclear, our ability to generate iPSCs from the CD4-negative cell population indicates that $CD4^+$ T cells or monocytes are not essential for reprogramming. CD4 depletion did not compromise reprogramming and was included solely to minimize proviral carryover. Notably, most lines exhibited a normal 46, XY karyotype, confirming genetic stability. However, nine lines presented chromosomal abnormalities, a common occurrence in iPSC reprogramming (Gore et al., 2011): we could not find correlations between genetic abnormality and features such as cryopreservation time, donor, or clinical phenotype. All lines demonstrated typical iPSC morphology, robust expression of canonical pluripotency markers (OCT-4 and NANOG), and passed the three-germ layer differentiation assay, confirming pluripotency.

We demonstrated the functional utility of the MACS-iPSC lines for HIV-1 studies by differentiating a subset into macrophages, an *in vivo* HIV-1 target (Koenig et al., 1986). Macrophages from two RPs and two ECs displayed characteristic morphology, expressed pan-macrophage markers, and were capable of phagocytosis at levels comparable to MDMs, underscoring the potential of MACS-iPSCs for generating

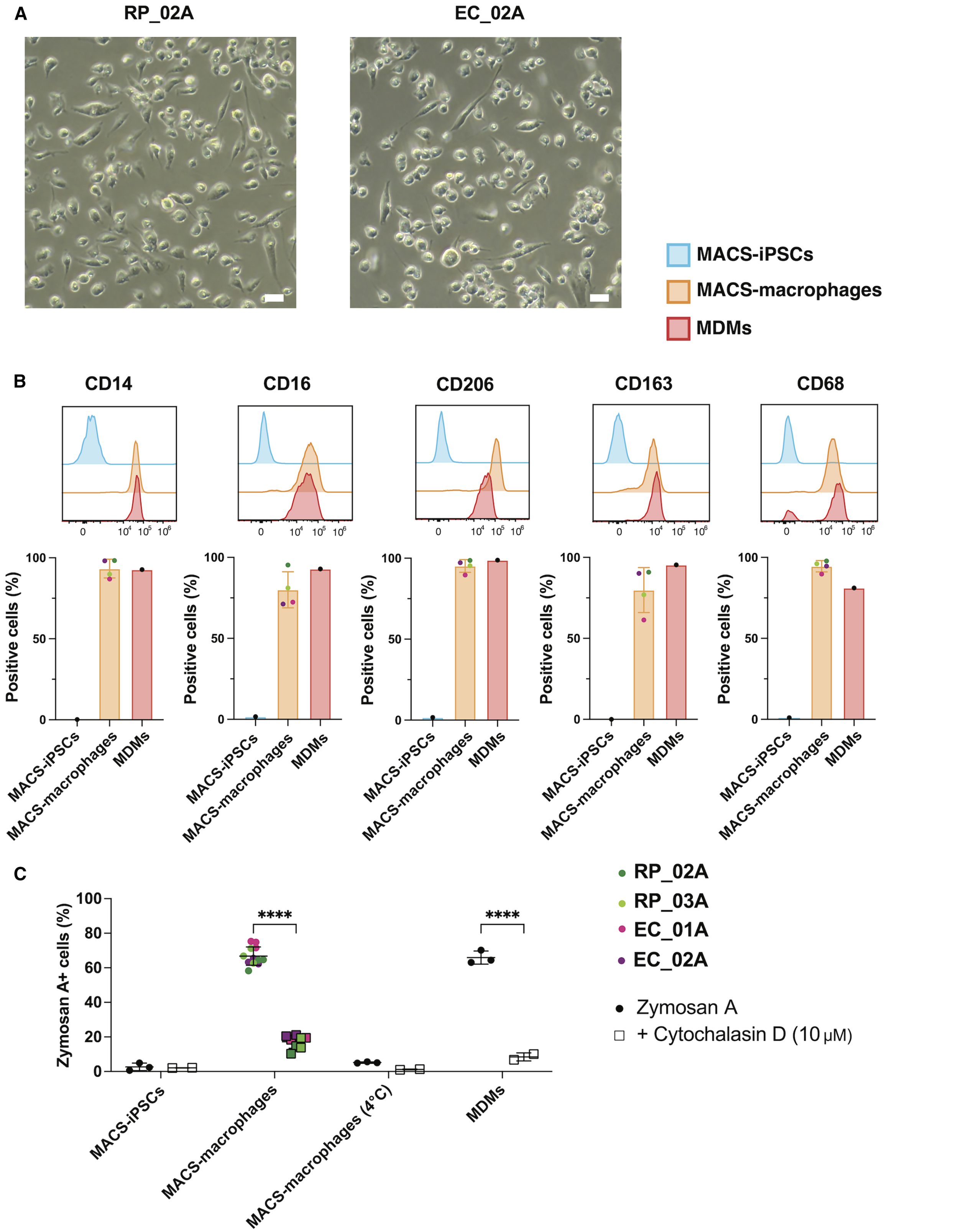

(legend on next page)

relevant cell types. Both undifferentiated MACS-iPSCs and derived macrophages supported HIV-1 infection, and macrophages are permissive to spreading HIV-1 replication. These results confirm that the MACS-iPSC resource can generate infection-competent target cells suitable for studying host-virus interactions. Although the present study was not designed or powered to evaluate relationships between donor clinical phenotype and *in vitro* susceptibility, establishing such links will be a valuable future application of this panel. By making all MACS-iPSC lines broadly accessible, we aim to enable future studies to address these lineage- and patient-specific questions.

Collectively, our findings affirm the quality and utility of this panel of iPSCs for a wide range of downstream applications in immunovirology, host-pathogen interaction studies, and regenerative medicine. While iPSCs have previously been used to model HIV-1 infection, this study, to the best of our knowledge, is the first to generate HIV-1 provirus-free iPSC lines from a clinically diverse and extensively phenotyped cohort of individuals before the ART era. Prior work has shown the feasibility of reprogramming PBMCs from HIV-1-infected individuals on ART (Teque et al., 2020; Ye et al., 2020), reflecting a context distinct from natural clinical trajectory. Our MACS-derived panel of iPSCs therefore provides a unique and complementary platform for HIV-1 research, especially in the context of natural infection in the absence of ART. However, a recognized limitation of iPSC-derived cells is their fetal-like phenotype and lack of aging-associated features (Horvath and Raj, 2018; Mertens et al., 2016), which may affect their ability to fully model HIV-1 pathogenesis.

Beyond the HIV-1 field, our iPSC panel holds broad potential for investigating host susceptibility and responses to a range of other pathogens, particularly those that frequently co-infect or act as opportunistic infections in HIV-1-positive individuals, such as *Mycobacterium tuberculosis* (Shankar et al., 2014), *Pneumocystis jirovecii* (Balaan, 1990; Vento et al., 1993), and *Salmonella enterica* (Feasey et al., 2012). While we demonstrated differentiation into macrophages, MACS-iPSCs could be used to generate multiple relevant lineages, such as $CD4^+$ T cells, microglia, and dendritic cells, providing additional avenues to investigate patient-specific responses to HIV-1 and dissect disease mechanisms in a lineage-specific manner (Fong et al., 2025; Min et al., 2023; Monkley et al., 2020). Moreover, coupled with recent advancements in stem cell technologies, including increasingly sophisticated three-dimensional organoid models that closely recapitulate native tissue architecture and function (Schutgens and Clevers, 2020), MACS-iPSC lines open up avenues to explore HIV-1 infection in relevant tissue microenvironments in a clinical phenotype-specific manner. Given the early and robust replication of HIV-1 in gut-associated lymphoid tissue (Brenchley et al., 2004), gut organoids derived from MACS-iPSCs may be especially informative for dissecting patient-specific pathobiology (Takahashi et al., 2018). Other models, such as brain organoids (Lancaster and Knoblich, 2014; Popova et al., 2021) and lymphoid organoids (Ramos et al., 2023), could further expand the platform's relevance to studying viral pathogenesis and persistence in difficult-to-access tissues. By enabling exploration of both cellular and tissue-level factors that shape infection and through open sharing of these lines with the research community, this iPSC panel offers a new and versatile resource for mechanistic studies of HIV-1 and beyond.

METHODS

Study subjects

Participants were men, primarily of White ethnicity, from MACS cohort (Chicago). They were followed every 6 months for behavioral data, HIV-1 serostatus, $CD4^+$/$CD8^+$ T cell counts, and, if infected, plasma HIV-1 RNA levels (quantitative reverse-transcription PCR [RT-qPCR], Roche). All subjects had equal access to care. Written informed consent for iPSC research was obtained in accordance with Northwestern University's human subjects' protection committee (Institutional Review Board protocol: STU00022906-CR0004). Original MACS identifiers were anonymized and replaced with study-specific codes reflecting clinical phenotypes.

Cell lines

HEK293T cells (ATCC-CRL-3216) were maintained in DMEM-GlutaMAX (Gibco) with 10% heat-inactivated FBS

Figure 4. Differentiation of MACS-iPSCs into macrophages across extreme clinical phenotypes
(A) Light microscopy images of representative macrophages derived from MACS-iPSC lines RP_02A and EC_02A. Scale bars, 50 μm.
(B) Representative histogram showing mean fluorescent intensity for MACS-iPSC macrophage line RP_02A (top) and quantification of expression levels of macrophage-associated markers CD14, CD16, CD206, CD163, and CD68, assessed by flow cytometry (bottom) in MACS-iPSC-derived macrophages compared to blood monocyte-derived macrophages (MDMs) and undifferentiated MACS-iPSCs.
(C) Flow cytometry quantification of GFP+ cells following uptake of zymosan A particles in undifferentiated MACS-iPSCs, MACS-iPSC-derived macrophages (four lines), and MDMs. Cells were either untreated, treated with 10 μM cytochalasin D, or incubated at 4°C to inhibit phagocytosis; $n = 3$ biological replicates for zymosan A and $n = 2$ biological replicates for cytochalasin D. Statistical test: two-way ANOVA with Fisher's LSD multiple comparisons test, $^{****}p < 0.0001$.

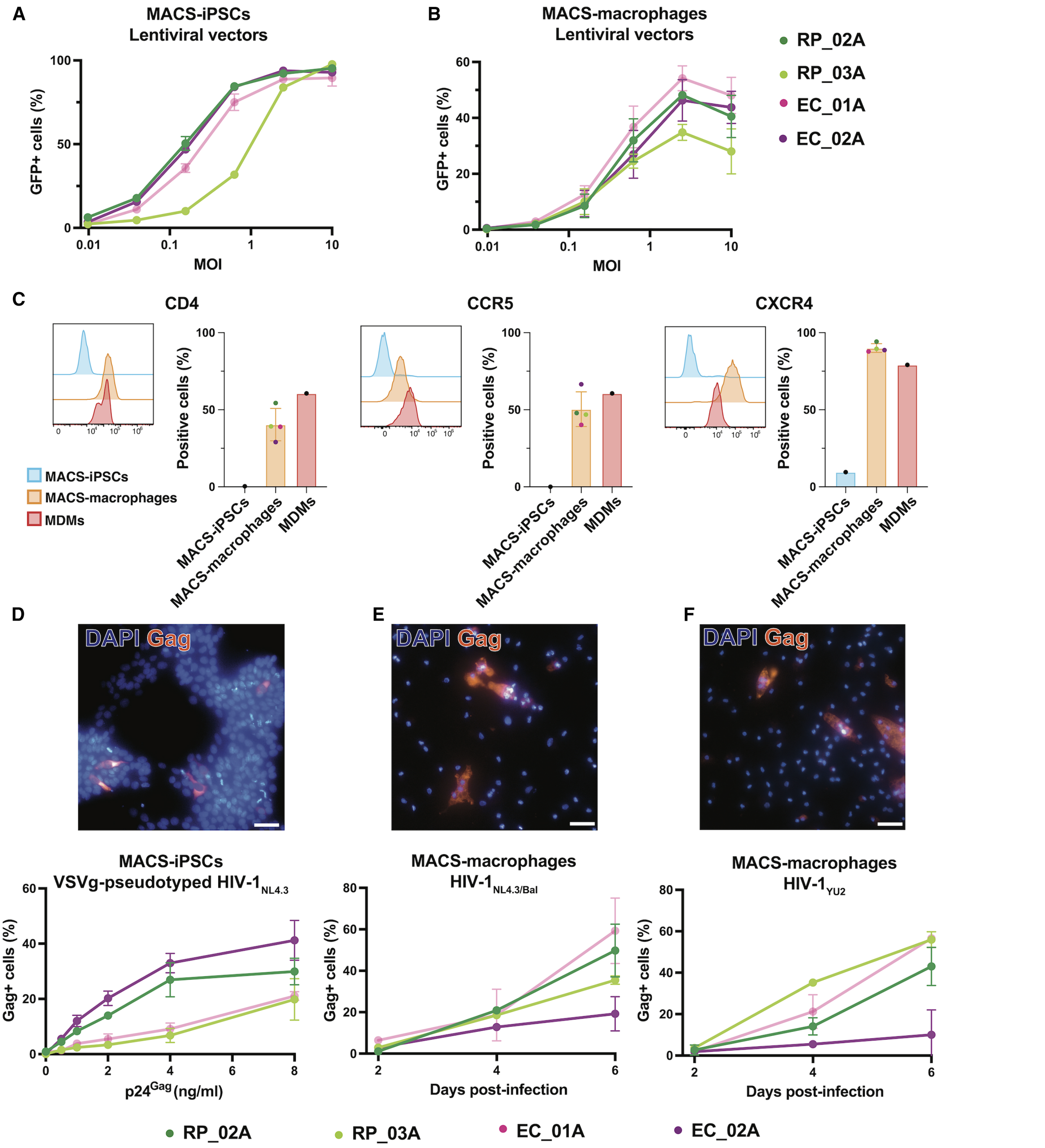

Figure 5. MACS-iPSCs and MACS-iPSC-derived macrophages can be successfully transduced by HIV-1-based lentiviral vectors and infected with full-length HIV-1

(A and B) Flow cytometry quantification of GFP+ cells in four lines of undifferentiated MACS-iPSCs (A) and MACS-derived macrophages (B) following transduction with increasing MOI of VSV-G-pseudotyped HIV-1-based lentiviral vectors expressing GFP.

(legend continued on next page)

and 1% P/S. ACH-2 (ARP-349) cells were cultured in RPMI-1640 with 2 mM L-glutamine (Gibco), 10% heat-inactivated fetal bovine serum (FBS, Sigma-Aldrich), 10 mM HEPES, 1× MEM non-essential amino acids, and 1% penicillin/streptomycin (P/S) (all from Gibco). iPSCs were maintained on vitronectin (Gibco, A14700)-coated plates in complete E8 medium (Gibco) with 1% P/S (E8+) and daily medium changes. All lines were routinely screened for mycoplasma (MycoAlert, Lonza) and maintained at 37°C, 5% CO_2. Newly generated iPSCs were cultured at 38.5°C for 5 days to promote Sendai vectors clearance.

PBMC isolation, sorting, and activation

PBMCs were isolated from venous blood obtained from MACS participants or healthy donors via the King's College London Infectious Diseases BioBank (ethics reference MM2-220518), under overall permission from the Southampton and South West Hampshire Research Ethics Committee (REC reference 19/SC/0232). PBMCs were separated by density gradient centrifugation using SepMate and Lymphoprep (STEMCELL Technologies). $CD4^+$ cells were removed by magnetic depletion (CD4 MicroBeads, Miltenyi Biotec).

iPSC generation and culturing

PBMCs (fresh or cryopreserved), with or without $CD4^+$ cell depletion, were reprogrammed using the CytoTune-iPS 2.0 Sendai Reprogramming Kit (Invitrogen). Four days prior to transduction, PBMCs were seeded at 5×10^5 cells/mL in StemPro-34 SFM (Gibco) supplemented with 2 mM L-glutamine, 100 ng/mL SCF, 100 ng/mL FLT3 ligand, 20 ng/mL IL-3, 20 ng/mL IL-6 (all from Thermo Fisher Scientific), and 1% P/S. Medium was partially replaced daily. For reprogramming, 3×10^5 PBMCs were transduced with Sendai vectors encoding KOS, KLF4, and c-MYC (MOIs 5, 3, and 5) by spinfection (1,000 × *g*, 30 min, RT). After overnight incubation, cells were washed and returned to PBMC medium. After an additional 48 h, cells were seeded onto vitronectin-coated plates in cytokine-free StemPro-34 SFM medium at densities of 10^4–10^5 cells/well. Two days later, cultures were transitioned to E8+ by gradual replacement, followed by daily changes. Emerging colonies with typical iPSC morphology were manually picked and expanded. Cells were passaged using gentle cell dissociation reagent (GDCR, STEMCELL Technologies) every 4–5 days. All experiments used passages 15–30. iPSCs were cryopreserved in 90% KnockOut Serum Replacement (Gibco) and 10% DMSO and thawed in E8+ with RevitaCell (Gibco). All MACS-iPSC lines are banked at King's College London Biobank under unique MACS ID identifiers. Quality control of MACS-iPSCs is detailed in the supplemental methods.

Macrophage differentiation

MACS-derived macrophages were generated using a modified version of van Wilgenburg et al. (2013). iPSCs (70%–90% confluence) were dissociated with TrypLE Express (Gibco) and seeded at 1.25×10^5 cells/well in ultra-low attachment 96-well plates (Corning Costar) in E8+ with 50 ng/mL BMP-4 (Bio-Techne, 314-BP), 50 ng/mL VEGF (Bio-Techne, 293-VE), 20 ng/mL SCF (Bio-Techne, 11010-SC), and 1 μM Y-27632 (Enzo Life Sciences, ALX-270-333) to form embryoid bodies (EBs). After 48 h, medium was refreshed without Y-27632. On day 4, EBs were transferred to 0.1% gelatine-coated dishes and cultured in X-VIVO 15 (Lonza) with 1% GlutaMAX (Gibco), 1% P/S, 0.055 mM β-mercaptoethanol (Gibco), 100 ng/mL M-CSF (Bio-Techne, 216-MC), and 25 ng/mL IL-3 (Bio-Techne, 203-IL). Myeloid precursors released into the supernatant were collected and differentiated in RPMI-1640 with 100 ng/mL M-CSF for 5–7 days. MDMs were generated from healthy-donor PBMCs by $CD14^+$ magnetic enrichment (CD14 MicroBeads, Miltenyi Biotec) and cultured in RPMI-1640 medium with 10% autologous human serum, 1% P/S, and 100 ng/mL M-CSF for 7 days. Quality control of macrophages derived from MACS-iPSCs is detailed in the supplemental methods.

Lentivirus vector and full-length HIV-1

For iPSC transductions, single cells obtained with GDCR were plated on vitronectin-coated plates and transduced with lentiviral vectors at a starting MOI of 10. Cells were fixed 48 h later (2% PFA) and analyzed for GFP by flow cytometry. For HIV-1 infection, iPSCs were incubated with

(C) Representative histogram showing mean fluorescent intensity of CD4, CCR5, and CXCR4 in MACS-iPSC macrophage line RP_02A (left) and quantification of receptor and co-receptor expression in MACS-iPSC-derived macrophages, compared to blood monocyte-derived macrophages (MDMs) and undifferentiated MACS-iPSCs (right).
(D) Representative immunofluorescence image (top) and quantification of Gag+ cells (bottom) in four undifferentiated MACS-iPSC lines infected with increasing concentrations of VSV-G-pseudotyped full-length HIV-1_{NL4-3}, 48 h post-infection, using high-throughput microscopy.
(E) Representative immunofluorescence image (top) and quantification of Gag+ cells (bottom) in four MACS-iPSC-derived macrophage lines infected with 1.9 ng/μL Gag HIV-$1_{NL4-3/BaL}$ on days 2, 4, and 6 post-infection, using high-throughput microscopy.
(F) Representative immunofluorescence image (top) and quantification of Gag+ cells (bottom) in four MACS-iPSC-derived macrophage lines infected with 2.4 ng/μL Gag HIV-1_{YU2} on days 2, 4, and 6, using high-throughput microscopy. Graphs represent the mean ± SD of $n = 4$ biological replicates per cell line.

VSV-G-pseudotyped HIV-$1_{NL4.3}$ (8 ng/μL $p24^{Gag}$). Viral input was removed after 24 h, and cells were fixed at 48 h for Gag staining. MACS-derived macrophages were transduced with a lentiviral vector at a starting MOI of 25 and analyzed 72 h later. For HIV-1 infection, cells were incubated with HIV-$1_{NL4.3/Bal}$ or HIV-1_{YU2} (190 ng/μL or 240 ng/μL $p24^{Gag}$) for 6 h, washed, and fixed at days 2, 4, and 6. The production of particles and quantification of infection are detailed in the supplemental methods.

Statistical tests

Statistical tests are indicated in figure legends. Analyses were performed using two-way ANOVA, with Fisher's LSD multiple comparisons test. Significance is indicated as $^{****}p < 0.0001$.

RESOURCE AVAILABILITY

Lead contact

Requests for further information, resources, and reagents should be directed to the lead contact, Luis Apolonia (luis.apolonia@kcl.ac.uk).

Materials availability

All MACS-iPSC lines generated in this study are available and will be supplied under a materials transfer agreement from the corresponding authors upon request.

Data and code availability

All data generated in this study adhere to FAIR (Findable, Accessible, Interoperable, and Reusable) principles, and data sharing respects CARE (Collective Benefit, Authority to Control, Responsibility, and Ethics) principles for human-derived materials. All raw data and associated processed datasets, including characterization data for all MACS-iPSC lines, are available from the lead contact upon reasonable request.

ACKNOWLEDGMENTS

We thank Fiona Watt and Subhankar Mukhopadhyay for helpful discussions and Ruta Meleckyte and Stelios Papaioannou for the provision of reagents. The work was funded by a Medical Research Council program grant (MR/S023747/1to M.H.M., L.A., D.D., and A.V.), the Wellcome Trust (222433/Z/21/Z to M.H.M.), and National Institutes of Health grants (U01AI035039 and U01HL146240 to S.M.W.). N.A. was funded by the Wellcome Trust PhD program in Cell Therapies and Regenerative Medicine (108874/Z/15/Z). S.A. was supported by a Medical Research Council – King's College London Doctoral Training Partnership in Biomedical Sciences industrial Collaborative Award in Science & Engineering (iCASE) in partnership with Orchard Therapeutics (MR/R015643/1). D.C. was supported by a Biotechnology and Biological Sciences Research Council CASE in partnership with GlaxoSmithKline (BB/V509632/1). This work, including the high-throughput screening facility "Stem Cell Hotel," was supported by the Department of Health via a National Institute for Health Research comprehensive Biomedical Research Centre award to Guy's and St Thomas' NHS Foundation Trust in partnership with King's College London and King's College Hospital NHS Foundation Trust.

AUTHOR CONTRIBUTIONS

N.A. and S.A. performed the experiments. D.C. contributed to karyotyping analyses. N.K. optimized the iPSC infection pipeline. L.F. and T.W. provided support for Operetta high-content analysis. P.A.O., E.-Y.K., and S.M.W. facilitated access to MACS samples and associated data. D.D. and A.V. contributed to project conception. M.H.M. and L.A. conceived and directed the study. N.A., M.H.M., and L.A. wrote the manuscript with input from all authors.

DECLARATION OF INTERESTS

D.D. holds a visiting position at King's College London and at the Cambridge Stem Cell Institute, and he is a co-founder of Migration Biotherapeutics and an independent partner of Hoya Consulting.

SUPPLEMENTAL INFORMATION

Supplemental information can be found online at https://doi.org/10.1016/j.stemcr.2025.102786.

Received: September 8, 2025
Revised: December 22, 2025
Accepted: December 22, 2025
Published: January 22, 2026

REFERENCES

Alizon, S., von Wyl, V., Stadler, T., Kouyos, R.D., Yerly, S., Hirschel, B., Böni, J., Shah, C., Klimkait, T., Furrer, H., et al. (2010). Phylogenetic Approach Reveals That Virus Genotype Largely Determines HIV Set-Point Viral Load. PLoS Pathog. *6*, e1001123. https://doi.org/10.1371/journal.ppat.1001123.

An, P., and Winkler, C.A. (2010). Host genes associated with HIV/AIDS: advances in gene discovery. Trends Genet. *26*, 119–131. https://doi.org/10.1016/j.tig.2010.01.002.

Avior, Y., Lezmi, E., Eggan, K., and Benvenisty, N. (2021). Cancer-Related Mutations Identified in Primed Human Pluripotent Stem Cells. Cell Stem Cell *28*, 10–11. https://doi.org/10.1016/j.stem.2020.11.013.

Balaan, M.R. (1990). Pneumocystis carinii pneumonia. W. V. Med. J. *86*, 561–565.

Ban, H., Nishishita, N., Fusaki, N., Tabata, T., Saeki, K., Shikamura, M., Takada, N., Inoue, M., Hasegawa, M., Kawamata, S., and Nishikawa, S.I. (2011). Efficient generation of transgene-free human induced pluripotent stem cells (iPSCs) by temperature-sensitive Sendai virus vectors. Proc. Natl. Acad. Sci. USA *108*, 14234–14239. https://doi.org/10.1073/pnas.1103509108.

Barkan, S.E., Melnick, S.L., Preston-Martin, S., Weber, K., Kalish, L.A., Miotti, P., Young, M., Greenblatt, R., Sacks, H., and Feldman, J. (1998). The Women's Interagency HIV Study. WIHS Collaborative Study Group. Epidemiology *9*, 117–125.

Blanquart, F., Wymant, C., Cornelissen, M., Gall, A., Bakker, M., Bezemer, D., Hall, M., Hillebregt, M., Ong, S.H., Albert, J., et al. (2017). Viral genetic variation accounts for a third of variability in HIV-1 set-point viral load in Europe. PLoS Biol. *15*, e2001855. https://doi.org/10.1371/journal.pbio.2001855.

Boshoff, C., and Weiss, R. (2002). AIDS-related malignancies. Nat. Rev. Cancer *2*, 373–382. https://doi.org/10.1038/NRC797.

Brenchley, J.M., Schacker, T.W., Ruff, L.E., Price, D.A., Taylor, J.H., Beilman, G.J., Nguyen, P.L., Khoruts, A., Larson, M., Haase, A.T., and Douek, D.C. (2004). CD4+ T cell depletion during all stages of HIV disease occurs predominantly in the gastrointestinal tract. J. Exp. Med. *200*, 749–759. https://doi.org/10.1084/JEM.20040874.

Chen, G., Gulbranson, D.R., Hou, Z., Bolin, J.M., Ruotti, V., Probasco, M.D., Smuga-Otto, K., Howden, S.E., Diol, N.R., Propson, N.E., et al. (2011). Chemically defined conditions for human iPSC derivation and culture. Nat. Methods *8*, 424–429. https://doi.org/10.1038/nmeth.1593.

D'Antonio, M., Benaglio, P., Jakubosky, D., Greenwald, W.W., Matsui, H., Donovan, M.K.R., Li, H., Smith, E.N., D'Antonio-Chronowska, A., and Frazer, K.A. (2018). Insights into the Mutational Burden of Human Induced Pluripotent Stem Cells from an Integrative Multi-Omics Approach. Cell Rep. *24*, 883–894. https://doi.org/10.1016/j.celrep.2018.06.091.

Dean, M., Carrington, M., Winkler, C., Huttley, G.A., Smith, M.W., Allikmets, R., Goedert, J.J., Buchbinder, S.P., Vittinghoff, E., Gomperts, E., et al. (1996). Genetic Restriction of HIV-1 Infection and Progression to AIDS by a Deletion Allele of the *CKR5* Structural Gene. Science *273*, 1856–1862. https://doi.org/10.1126/science.273.5283.1856.

D'Souza, G., Bhondoekhan, F., Benning, L., Margolick, J.B., Adedimeji, A.A., Adimora, A.A., Alcaide, M.L., Cohen, M.H., Detels, R., Friedman, M.R., et al. (2021). Characteristics of the MACS/WIHS Combined Cohort Study: Opportunities for Research on Aging With HIV in the Longest US Observational Study of HIV. Am. J. Epidemiol. *190*, 1457–1475. https://doi.org/10.1093/aje/kwab050.

Egger, M., Hirschel, B., Francioli, P., Sudre, P., Wirz, M., Flepp, M., Rickenbach, M., Malinverni, R., Vernazza, P., and Battegay, M. (1997). Impact of new antiretroviral combination therapies in HIV infected patients in Switzerland: prospective multicentre study. Swiss HIV Cohort Study. BMJ *315*, 1194–1199. https://doi.org/10.1136/bmj.315.7117.1194.

Eisinger, R.W., Dieffenbach, C.W., and Fauci, A.S. (2019). HIV Viral Load and Transmissibility of HIV Infection: Undetectable Equals Untransmittable. JAMA *321*, 451–452. https://doi.org/10.1001/JAMA.2018.21167.

Feasey, N.A., Dougan, G., Kingsley, R.A., Heyderman, R.S., and Gordon, M.A. (2012). Invasive non-typhoidal salmonella disease: an emerging and neglected tropical disease in Africa. Lancet *379*, 2489–2499. https://doi.org/10.1016/S0140-6736(11)61752-2.

Fong, H., Mendel, M., Jascur, J., Najmi, L., Kim, K., Lew, G., Garimalla, S., Schock, S., Hu, J., Villegas, A.G., et al. (2025). A serum- and feeder-free system to generate CD4 and regulatory T cells from human iPSCs. Stem Cell. *43*, sxaf001. https://doi.org/10.1093/stmcls/sxaf001.

Fraser, C., Lythgoe, K., Leventhal, G.E., Shirreff, G., Hollingsworth, T.D., Alizon, S., and Bonhoeffer, S. (2014). Virulence and Pathogenesis of HIV-1 Infection: An Evolutionary Perspective. Science *343*, 1243727. https://doi.org/10.1126/science.1243727.

Fusaki, N., Ban, H., Nishiyama, A., Saeki, K., and Hasegawa, M. (2009). Efficient induction of transgene-free human pluripotent stem cells using a vector based on Sendai virus, an RNA virus that does not integrate into the host genome. Proc. Jpn. Acad. Ser. B Phys. Biol. Sci. *85*, 348–362. https://doi.org/10.2183/pjab.85.348.

Gore, A., Li, Z., Fung, H.-L., Young, J.E., Agarwal, S., Antosiewicz-Bourget, J., Canto, I., Giorgetti, A., Israel, M.A., Kiskinis, E., et al. (2011). Somatic coding mutations in human induced pluripotent stem cells. Nature *471*, 63–67. https://doi.org/10.1038/nature09805.

Gutbier, S., Wanke, F., Dahm, N., Rümmelin, A., Zimmermann, S., Christensen, K., Köchl, F., Rautanen, A., Hatje, K., Geering, B., et al. (2020). Large-scale production of human IPSC-derived macrophages for drug screening. Int. J. Mol. Sci. *21*, 4808. https://doi.org/10.3390/ijms21134808.

Haake, K., Neehus, A.-L., Buchegger, T., Kühnel, M.P., Blank, P., Philipp, F., Oleaga-Quintas, C., Schulz, A., Grimley, M., Goethe, R., et al. (2020). Patient iPSC-Derived Macrophages to Study Inborn Errors of the IFN-γ Responsive Pathway. Cells *9*, 483. https://doi.org/10.3390/cells9020483.

Horvath, S., and Raj, K. (2018). DNA methylation-based biomarkers and the epigenetic clock theory of ageing. Nat. Rev. Genet. *19*, 371–384. https://doi.org/10.1038/s41576-018-0004-3.

Huang, Y., Paxton, W.A., Wolinsky, S.M., Neumann, A.U., Zhang, L., He, T., Kang, S., Ceradini, D., Jin, Z., Yazdanbakhsh, K., et al. (1996). The role of a mutant CCR5 allele in HIV–1 transmission and disease progression. Nat. Med. *2*, 1240–1243. https://doi.org/10.1038/nm1196-1240.

Jiang, Y., Chen, O., Cui, C., Zhao, B., Han, X., Zhang, Z., Liu, J., Xu, J., Hu, Q., Liao, C., and Shang, H. (2013). KIR3DS1/L1 and HLA-Bw4-80I are associated with HIV disease progression among HIV typical progressors and long-term nonprogressors. BMC Infect. Dis. *13*, 405. https://doi.org/10.1186/1471-2334-13-405.

Kambal, A., Mitchell, G., Cary, W., Gruenloh, W., Jung, Y., Kalomoiris, S., Nacey, C., McGee, J., Lindsey, M., Fury, B., et al. (2011). Generation of HIV-1 Resistant and Functional Macrophages From Hematopoietic Stem Cell–derived Induced Pluripotent Stem Cells. Mol. Ther. *19*, 584–593. https://doi.org/10.1038/mt.2010.269.

Kaslow, R.A., Ostrow, D.G., Detels, R., Phair, J.P., Polk, B.F., and Rinaldo, C.R. (1987). The Multicenter AIDS Cohort Study: rationale, organization, and selected characteristics of the participants. Am. J. Epidemiol. *126*, 310–318. https://doi.org/10.1093/aje/126.2.310.

Kaslow, R.A., Carrington, M., Apple, R., Park, L., Muñoz, A., Saah, A.J., Goedert, J.J., Winkler, C., O'Brien, S.J., Rinaldo, C., et al. (1996). Influence of combinations of human major histocompatibility complex genes on the course of HIV–1 infection. Nat. Med. *2*, 405–411. https://doi.org/10.1038/nm0496-405.

Koenig, S., Gendelman, H.E., Orenstein, J.M., Dal Canto, M.C., Pezeshkpour, G.H., Yungbluth, M., Janotta, F., Aksamit, A., Martin, M.A., and Fauci, A.S. (1986). Detection of AIDS virus in macrophages in brain tissue from AIDS patients with encephalopathy. Science *233*, 1089–1093. https://doi.org/10.1126/SCIENCE.3016903.

Kulkarni, S., Lied, A., Kulkarni, V., Rucevic, M., Martin, M.P., Walker-Sperling, V., Anderson, S.K., Ewy, R., Singh, S., Nguyen, H., et al. (2019). CCR5AS lncRNA variation differentially regulates CCR5, influencing HIV disease outcome. Nat. Immunol. *20*, 824–834. https://doi.org/10.1038/s41590-019-0406-1.

Lancaster, M.A., and Knoblich, J.A. (2014). Generation of cerebral organoids from human pluripotent stem cells. Nat. Protoc. *9*, 2329–2340. https://doi.org/10.1038/nprot.2014.158.

Liu, R., Paxton, W.A., Choe, S., Ceradini, D., Martin, S.R., Horuk, R., MacDonald, M.E., Stuhlmann, H., Koup, R.A., and Landau, N.R. (1996). Homozygous Defect in HIV-1 Coreceptor Accounts for Resistance of Some Multiply-Exposed Individuals to HIV-1 Infection. Cell *86*, 367–377. https://doi.org/10.1016/S0092-8674(00)80110-5.

McLaren, P.J., and Carrington, M. (2015). The impact of host genetic variation on infection with HIV-1. Nat. Immunol. *16*, 577–583. https://doi.org/10.1038/ni.3147.

McLaren, P.J., and Fellay, J. (2021). HIV-1 and human genetic variation. Nat. Rev. Genet. *22*, 645–657. https://doi.org/10.1038/s41576-021-00378-0.

Mertens, J., Marchetto, M.C., Bardy, C., and Gage, F.H. (2016). Evaluating cell reprogramming, differentiation and conversion technologies in neuroscience. Nat. Rev. Neurosci. *17*, 424–437. https://doi.org/10.1038/nrn.2016.46.

Min, A.K., Javidfar, B., Missall, R., Doanman, D., Durens, M., Graziani, M., Mordelt, A., Marro, S.G., de Witte, L., Chen, B.K., et al. (2023). HIV-1 infection of genetically engineered iPSC-derived central nervous system-engrafted microglia in a humanized mouse model. J. Virol. *97*, e0159523. https://doi.org/10.1128/jvi.01595-23.

Monkley, S., Krishnaswamy, J.K., Göransson, M., Clausen, M., Meuller, J., Thörn, K., Hicks, R., Delaney, S., and Stjernborg, L. (2020). Optimised generation of iPSC-derived macrophages and dendritic cells that are functionally and transcriptionally similar to their primary counterparts. PLoS One *15*, e0243807. https://doi.org/10.1371/journal.pone.0243807.

Ou, C.Y., Kwok, S., Mitchell, S.W., Mack, D.H., Sninsky, J.J., Krebs, J.W., Feorino, P., Warfield, D., and Schochetman, G. (1988). DNA amplification for direct detection of HIV-1 in DNA of peripheral blood mononuclear cells. Science *239*, 295–297. https://doi.org/10.1126/science.3336784.

Picker, L.J. (2006). Immunopathogenesis of acute AIDS virus infection. Curr. Opin. Immunol. *18*, 399–405. https://doi.org/10.1016/J.COI.2006.05.001.

Popova, G., Soliman, S.S., Kim, C.N., Keefe, M.G., Hennick, K.M., Jain, S., Li, T., Tejera, D., Shin, D., Chhun, B.B., et al. (2021). Human microglia states are conserved across experimental models and regulate neural stem cell responses in chimeric organoids. Cell Stem Cell *28*, 2153–2166.e6. https://doi.org/10.1016/j.stem.2021.08.015.

Quinn, T.C., Wawer, M.J., Sewankambo, N., Serwadda, D., Li, C., Wabwire-Mangen, F., Meehan, M.O., Lutalo, T., and Gray, R.H. (2000). Viral load and heterosexual transmission of human immunodeficiency virus type 1. Rakai Project Study Group. N. Engl. J. Med. *342*, 921–929. https://doi.org/10.1056/NEJM200003303421303.

Ramos, S.A., Armitage, L.H., Morton, J.J., Alzofon, N., Handler, D., Kelly, G., Homann, D., Jimeno, A., and Russ, H.A. (2023). Generation of functional thymic organoids from human pluripotent stem cells. Stem Cell Rep. *18*, 829–840. https://doi.org/10.1016/j.stemcr.2023.02.013.

Sabin, C.A., and Lundgren, J.D. (2013). The natural history of HIV infection. Curr. Opin. HIV AIDS *8*, 311–317. https://doi.org/10.1097/COH.0b013e328361fa66.

Schutgens, F., and Clevers, H. (2020). Human Organoids: Tools for Understanding Biology and Treating Diseases. Annu. Rev. Pathol. *15*, 211–234. https://doi.org/10.1146/annurev-pathmechdis-012419-032611.

Shankar, E.M., Vignesh, R., Ellegård, R., Barathan, M., Chong, Y.K., Bador, M.K., Rukumani, D.V., Sabet, N.S., Kamarulzaman, A., Velu, V., and Larsson, M. (2014). HIV- *Mycobacterium tuberculosis* co-infection: a ‘danger-couple model’ of disease pathogenesis. Pathog. Dis. *70*, 110–118. https://doi.org/10.1111/2049-632X.12108.

Shi, Y., Inoue, H., Wu, J.C., and Yamanaka, S. (2017). Induced pluripotent stem cell technology: A decade of progress. Nat. Rev. Drug Discov. *16*, 115–130. https://doi.org/10.1038/nrd.2016.245.

Silvestri, G., Sodora, D.L., Koup, R.A., Paiardini, M., O’Neil, S.P., McClure, H.M., Staprans, S.I., and Feinberg, M.B. (2003). Nonpathogenic SIV Infection of Sooty Mangabeys Is Characterized by Limited Bystander Immunopathology Despite Chronic High-Level Viremia. Immunity *18*, 441–452. https://doi.org/10.1016/S1074-7613(03)00060-8.

Sullivan, S., Stacey, G.N., Akazawa, C., Aoyama, N., Baptista, R., Bedford, P., Bennaceur Griscelli, A., Chandra, A., Elwood, N., Girard, M., et al. (2018). Quality Control Guidelines for Clinical-Grade Human Induced Pluripotent Stem Cell Lines. Regen. Med. *13*, 859–866. https://doi.org/10.2217/rme-2018-0095.

Takahashi, K., Tanabe, K., Ohnuki, M., Narita, M., Ichisaka, T., Tomoda, K., and Yamanaka, S. (2007). Induction of Pluripotent Stem Cells from Adult Human Fibroblasts by Defined Factors. Cell *131*, 861–872. https://doi.org/10.1016/j.cell.2007.11.019.

Takahashi, Y., Sato, S., Kurashima, Y., Yamamoto, T., Kurokawa, S., Yuki, Y., Takemura, N., Uematsu, S., Lai, C.-Y., Otsu, M., et al. (2018). A Refined Culture System for Human Induced Pluripotent Stem Cell-Derived Intestinal Epithelial Organoids. Stem Cell Rep. *10*, 314–328. https://doi.org/10.1016/j.stemcr.2017.11.004.

Teque, F., Ye, L., Xie, F., Wang, J., Morvan, M.G., Kan, Y.W., and Levy, J.A. (2020). Genetically-edited induced pluripotent stem cells derived from HIV-1-infected patients on therapy can give rise to immune cells resistant to HIV-1 infection. AIDS *34*, 1141–1149. https://doi.org/10.1097/QAD.0000000000002539.

International HIV Controllers Study, Pereyra, F., Jia, X., McLaren, P.J., Telenti, A., de Bakker, P.I.W., Walker, B.D., Ripke, S., Brumme, C.J., Pulit, S.L., et al. (2010). The Major Genetic Determinants of

HIV-1 Control Affect HLA Class I Peptide Presentation. Science *330*, 1551–1557. https://doi.org/10.1126/science.1195271.

Touloumi, G., Pantazis, N., Pillay, D., Paraskevis, D., Chaix, M.L., Bucher, H.C., Kücherer, C., Zangerle, R., Kran, A.M.B., and Porter, K.; CASCADE collaboration in EuroCoord (2013). Impact of HIV-1 subtype on CD4 count at HIV seroconversion, rate of decline, and viral load set point in European seroconverter cohorts. Clin. Infect. Dis. *56*, 888–897. https://doi.org/10.1093/CID/CIS1000.

UNAIDS. Global HIV & AIDS statistics — Fact sheet. https://www.unaids.org/en/resources/fact-sheet.

Vento, S., Di Perri, G., Garofano, T., Concia, E., and Bassetti, D. (1993). Pneumocystis carinii pneumonia during primary HIV-1 infection. Lancet *342*, 24–25. https://doi.org/10.1016/0140-6736(93)91884-O.

Wang, L., Chen, Y., Guan, C., Zhao, Z., Li, Q., Yang, J., Mo, J., Wang, B., Wu, W., Yang, X., et al. (2017). Using low-risk factors to generate non-integrated human induced pluripotent stem cells from urine-derived cells. Stem Cell Res. Ther. *8*, 245. https://doi.org/10.1186/s13287-017-0698-8.

Wei, Z., Bodnar, B., Zhao, R.-T., Xiao, Q., Saribas, S., Wang, X., Ho, W.-Z., and Hu, W. (2023). Human iPSC-derived brain organoids: A 3D mini-brain model for studying HIV infection. Exp. Neurol. *364*, 114386. https://doi.org/10.1016/j.expneurol.2023.114386.

van Wilgenburg, B., Browne, C., Vowles, J., and Cowley, S.A. (2013). Efficient, Long Term Production of Monocyte-Derived Macrophages from Human Pluripotent Stem Cells under Partly-Defined and Fully-Defined Conditions. PLoS One *8*, e71098. https://doi.org/10.1371/journal.pone.0071098.

Ye, L., Wang, J., Beyer, A.I., Teque, F., Cradick, T.J., Qi, Z., Chang, J.C., Bao, G., Muench, M.O., Yu, J., et al. (2014). Seamless modification of wild-type induced pluripotent stem cells to the natural CCR5Δ32 mutation confers resistance to HIV infection. Proc. Natl. Acad. Sci. USA *111*, 9591–9596. https://doi.org/10.1073/pnas.1407473111.

Ye, L., Wang, J., Teque, F., Xie, F., Tan, Y., Kan, Y.W., and Levy, J.A. (2020). Generation of HIV-1-infected patients' gene-edited induced pluripotent stem cells using feeder-free culture conditions. AIDS *34*, 1127–1139. https://doi.org/10.1097/QAD.0000000000002535.

Yu, J., Vodyanik, M.A., Smuga-Otto, K., Antosiewicz-Bourget, J., Frane, J.L., Tian, S., Nie, J., Jonsdottir, G.A., Ruotti, V., Stewart, R., et al. (2007). Induced Pluripotent Stem Cell Lines Derived from Human Somatic Cells. Science *318*, 1917–1920. https://doi.org/10.1126/science.1151526.

Zhang, H., Xue, C., Shah, R., Bermingham, K., Hinkle, C.C., Li, W., Rodrigues, A., Tabita-Martinez, J., Millar, J.S., Cuchel, M., et al. (2015). Functional Analysis and Transcriptomic Profiling of iPSC-Derived Macrophages and Their Application in Modeling Mendelian Disease. Circ. Res. *117*, 17–28. https://doi.org/10.1161/CIRCRESAHA.117.305860.

Stem Cell Reports, Volume *21*

Supplemental Information

Generation and characterization of iPSC models from HIV-1-positive individuals with divergent clinical outcomes

Nathalia Almeida, Sam Acors, Daniel Cox, Neophytos Kouphou, Lazaros Fotopoulos, Thomas Williams, Patricia A. Otto, Eun-Young Kim, Steven M. Wolinsky, Davide Danovi, Alessandra Vigilante, Michael H. Malim, and Luis Apolonia

Supplemental Figures

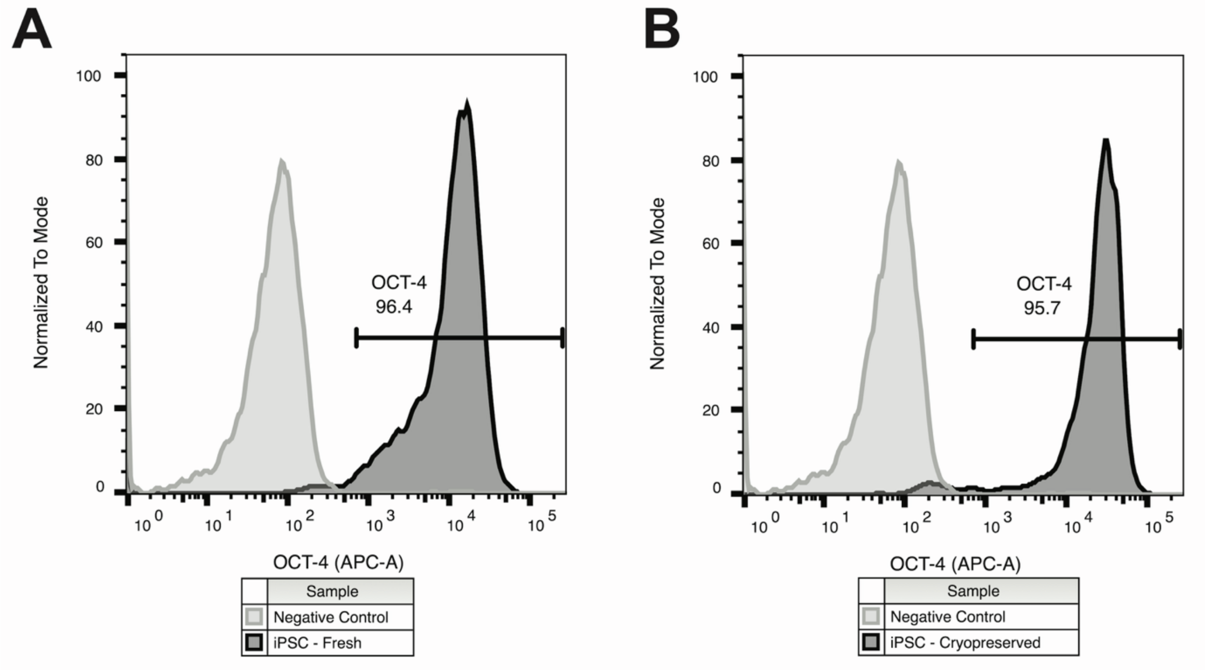

Figure S1. iPSCs generated from fresh or cryopreserved PBMCs express OCT-4. Histogram plots show OCT-4 expression in iPSC lines derived from either (A) fresh or (B) cryopreserved PBMCs, analysed by flow cytometry. iPSC populations (dark grey) are compared to a stained negative control line (HEK293T; light grey), confirming OCT-4 expression in both conditions.

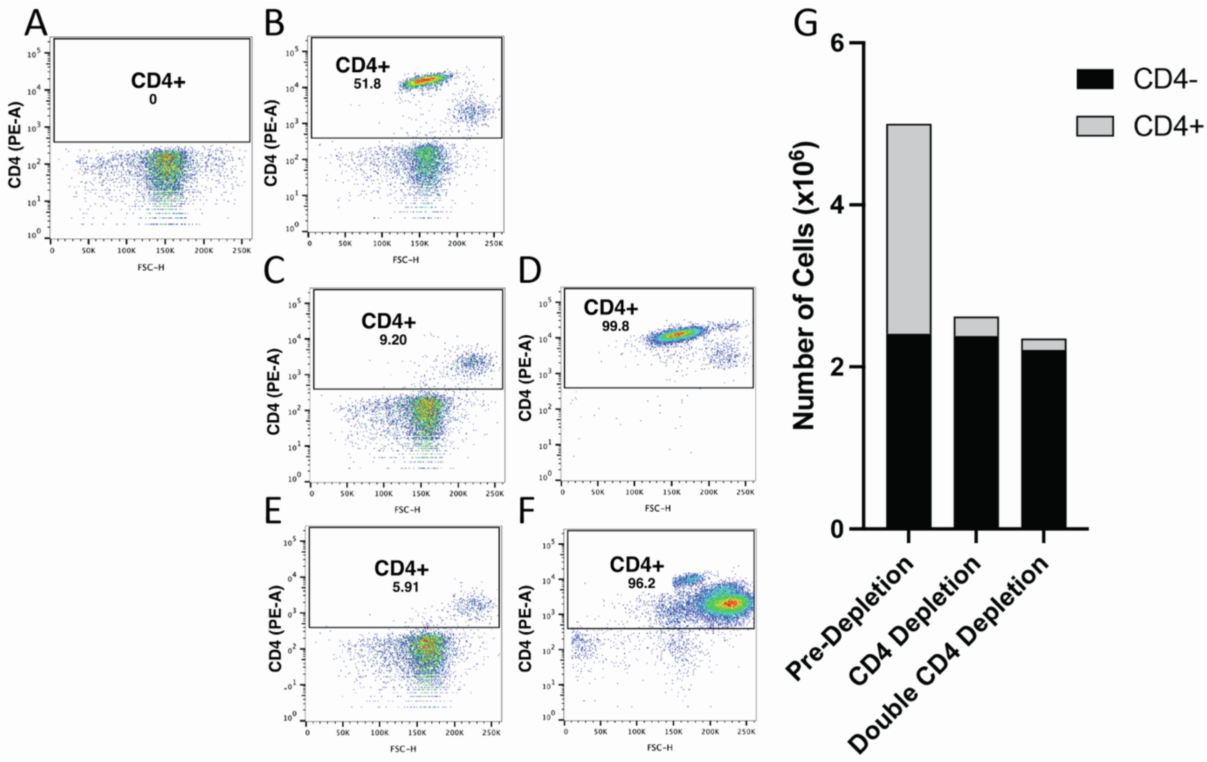

Figure S2. Optimisation of $CD4^+$ cell depletion using magnetic cell sorting.

Flow cytometry plots and a bar graph illustrating the efficiency of $CD4^+$ cell removal from a PBMC sample (healthy donor) following one or two rounds of magnetic depletion. (A) Unstained and (B) CD4-stained PBMCs prior to depletion. (C) $CD4^-$ and (D) $CD4^+$ fractions stained for CD4 after one round of depletion. (E) $CD4^-$ and (F) $CD4^+$ fractions stained after a second depletion round. (G) Total cell counts before depletion, after one round, and after two rounds, with $CD4^+$ cells shown in grey and $CD4^-$ cells in black.

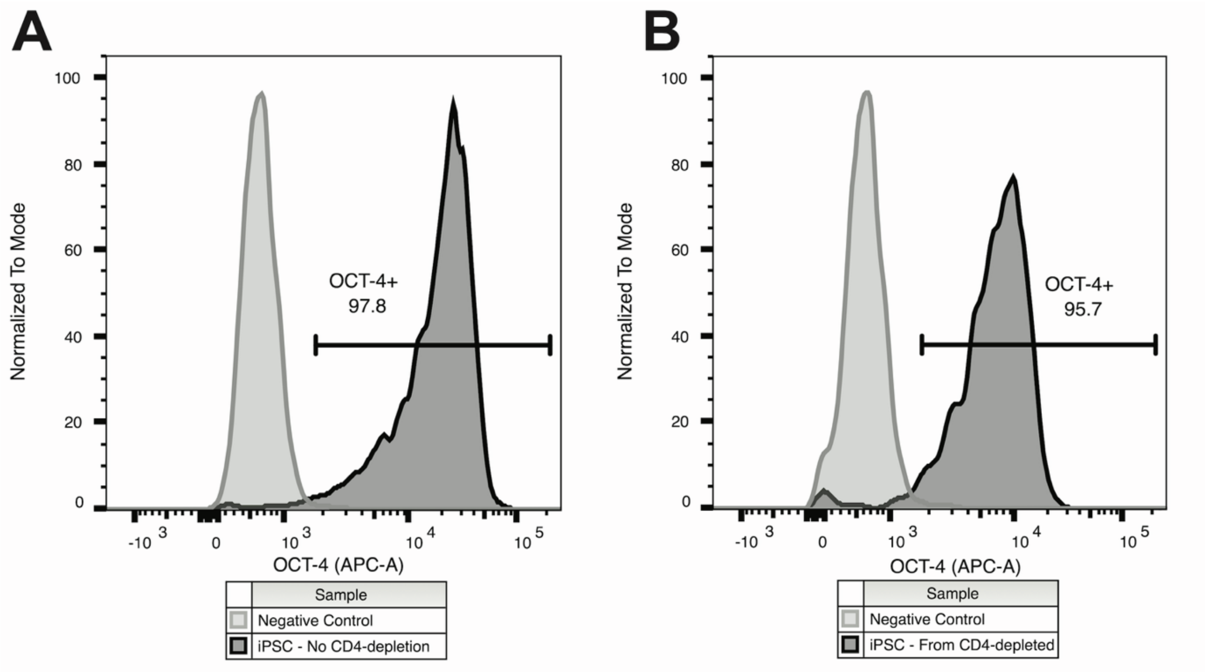

Figure S3. iPSCs generated from whole PBMCs or CD4-depleted PBMCs express OCT-4.

Histogram plots show OCT-4 expression in iPSC lines derived from either (A) whole PBMCs or (B) CD4-depleted PBMCs, analysed by flow cytometry. iPSC populations (dark grey) are compared with a stained negative control line (HEK293T; light grey), confirming robust OCT-4 expression under both reprogramming conditions.

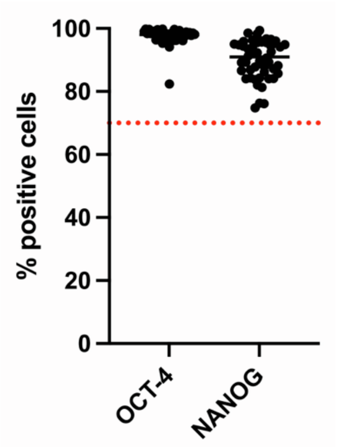

Figure S4. Pluripotency marker expression in MACS-iPSC lines, related to Figure 2.

Quantification of flow cytometry analysis of OCT-4 and NANOG expression across all 50 MACS-iPSC lines generated. The graph shows the percentage of cells positive for each marker, with a red dashed line indicating the 70% threshold required to define pluripotency.

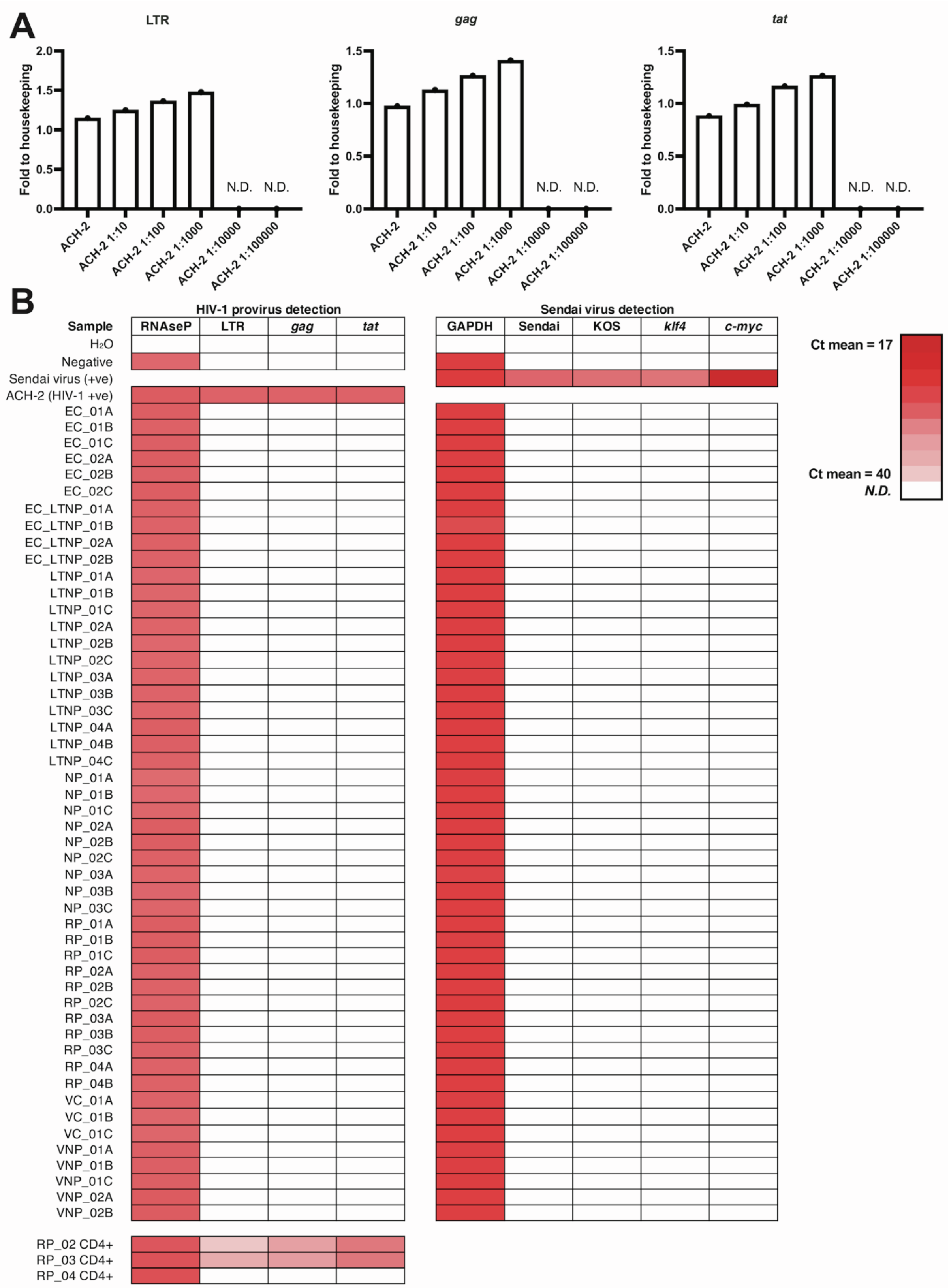

Figure S5. Detection of HIV-1 provirus and Sendai vectors on MACS-iPSC lines, related to Figure 2.

(A) Sensitivity of the HIV-1 proviral DNA qPCR assay. Genomic DNA from ACH-2 cells (one integrated provirus per cell) was serially diluted into HEK293T DNA and analysed by qPCR

targeting conserved regions with the 5' LTR, *gag*, and *tat*. RNaseP served as an internal genomic DNA control. Data are shown as fold change relative to RNaseP. N.D. indicates no detectable amplification. (B) Heatmap summarising qPCR screening of all MACS-iPSC lines for HIV-1 proviral sequences (5' LTR, *gag*, *tat*) and residual Sendai reprogramming vectors (Sendai, KOS, *Klf4*, *c-Myc*). Ct values are shown as a gradient from bright red (Ct = 17) to pale red (Ct = 40); N.D. indicates no detectable amplification. Positive controls included genomic RNA from the Sendai virus vector stock and genomic DNA from ACH-2 cells. Genomic DNA from the CD4+ PBMC fractions obtained during the depletion workflow are shown at the bottom.

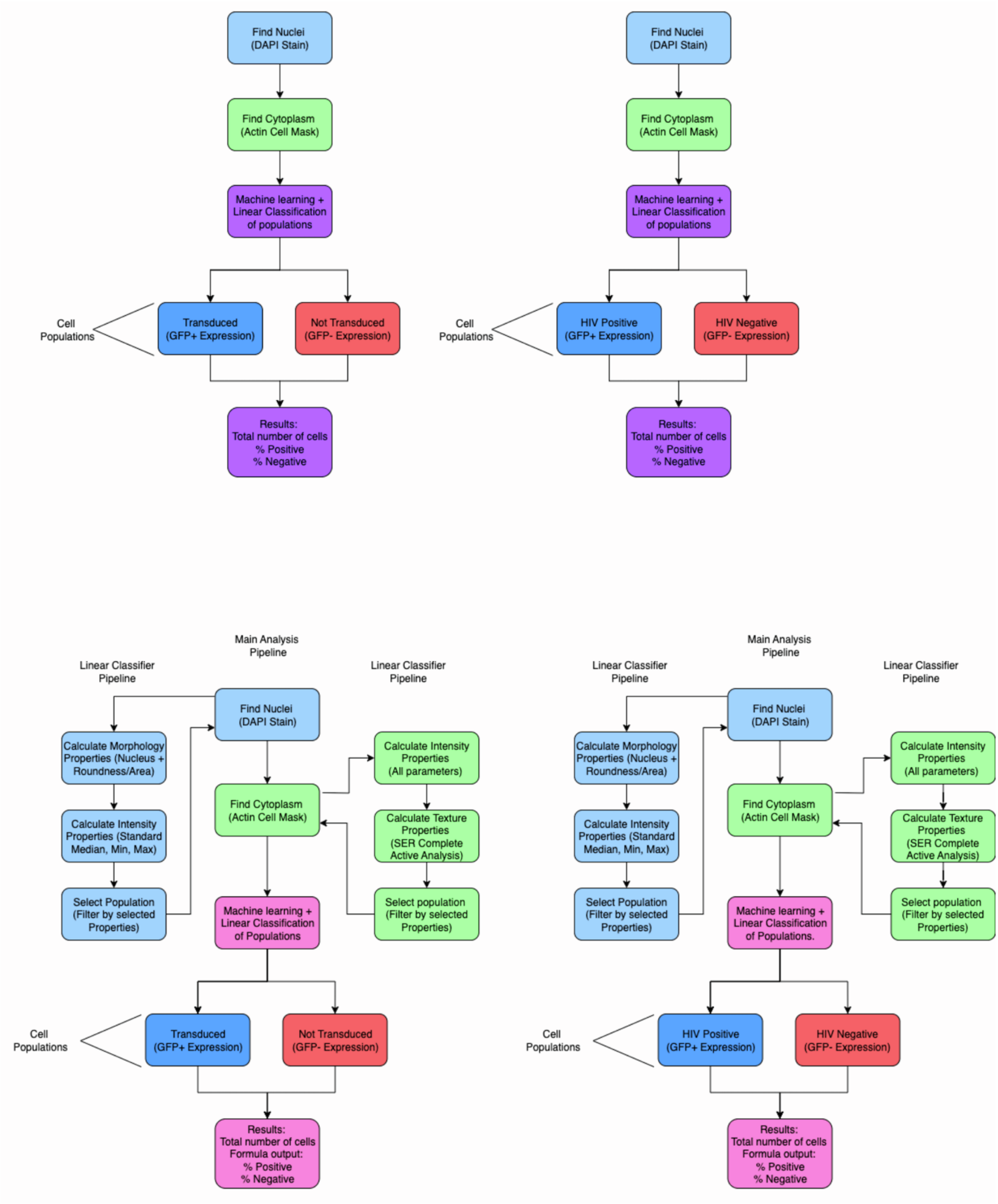

Figure S6. Diagram of Operetta algorithms to quantify transduction/infection. Harmony 4.9 custom pipelines to assess transduction with lentiviral vectors (left) or infection with full-length HIV-1 viruses (right).

Supplemental Tables

Table S1. Primers and probes used for the detection of HIV-1 provirus by qPCR, related to Figures 2 and S5.

Primer name	Primer sequence (5'-3')
HIV-1 LTR forward	CAGATGCTGCATATAAGCAGCTG
HIV-1 LTR reverse	GAGGGATCTCTAGNYACCAGAGT
HIV-1 LTR probe	FAM - CCTGTACTGGGTCTCTCTGG - TAMRA
HIV-1 *gag* forward	ATAATCCACCTATCCCAGTAGGAGAAAT
HIV-1 *gag* reverse	TTTGGTCCTTGTCTTATGTCCAGAATGC
HIV-1 *gag* probe	FAM - ATCCTGGGATTAAATAAAATAGTAAGAATGTATAGCCCTAC - TAMRA
HIV-1 *tat* forward	CCTGTGCCTCTTCAGCTAC
HIV-1 *tat* reverse	ATCCTTTCCAAGCCCTGTCT

Supplemental Methods

HIV-1 molecular clones, lentiviral vectors and production

The pNL4-3 provirus is a full-length, replication-competent HIV-1 molecular clone (Adachi et al., 1986). The CCR5-tropic pNL4-3$_{Bal}$ derivative contains the Bal env (Rasaiyaah et al., 2013). The pYU2 clone derives from the original HIV-1$_{YU2}$ isolate (Fouchier et al., 1997). Lentiviral vector plasmids included p8.91 (pCMV-ΔR8.91), pGFP (pHRsin.SFFV.eGFP), and pVSVg (pMD2.G), as described (Dull et al., 1998; Zufferey et al., 1997).

Lentiviral vectors were generated by co-transfecting HEK293T cells with p8.91, pGFP, and pVSV-G (1:1:0.125) using polyethyleneimine (PolySciences). VSV-G-pseudotyped HIV-1 was produced via co-transfection of pNL4.3 with pVSV-G (20:1). Full-length HIV-1$_{NL43/Bal}$ or HIV-1$_{YU2}$ stocks were produced similarly with the corresponding proviral plasmid. Supernatants were collected 48h post-transfection, filtered (0.45 μm), and concentrated by ultracentrifugation through 20% sucrose (150,000 × g, 75 min, 4 °C). Viral particles were quantified by p24^{Gag} AlphaLISA (PerkinElmer, AL291 C/F) or by titration on HEK293Ts cells.

Three-germ layer differentiation

Three-germ-layer differentiation was performed using the STEMdiff Trilineage Differentiation Kit (STEMCELL Technologies) according to the manufacturer's instructions. iPSCs were seeded onto Matrigel-coated 96-well plates, and, after 24h, cultured in the kit's lineage-specific media. Mesoderm, endoderm, and undifferentiated controls were fixed on day 4; ectoderm cultures were fixed on day 6.

G-banded karyotype analysis

G-banded karyotyping of MACS-iPSC lines (passages 15-30) was performed by Cell Guidance Systems following the provider's instructions.

Quantitative PCR

Genomic DNA and total RNA were extracted using the DNeasy Blood & Tissue Kit (Qiagen) and the Monarch Total RNA Miniprep Kit (New England Biolabs). cDNA was synthesised from 1 μg RNA using the High-Capacity cDNA Reverse Transcription Kit (Thermo Fisher Scientific). HIV-1 provirus was detected by qPCR targeting conserved 5'-LTR and *gag* regions (TaqMan, Thermo Fisher Scientific) and *tat* (PoweUp SYBR Green, Thermo Fisher Scientific) using primers listed in **Table S1**. Sendai virus (SeV) and reprogramming vectors (KOS, KLF4, c-MYC) were detected by TaqMan qPCR on cDNA using pre-designed primers (Thermo Fisher Scientific). Results are reported as mean Ct; samples with no amplification within 40 cycles were recorded as not determined (N.D.).

Flow cytometry

iPSCs were dissociated with GCDR, fixed, permeabilised with 0.1% Triton X-100 (Sigma-Aldrich), and blocked in 2% bovine serum albumin (BSA, Sigma-Aldrich). Cells were stained with PE-conjugated anti OCT-4 (1:100, BioLegend, 653704) or NANOG (1:100, Invitrogen, PA5-46891) in 2% BSA/PBS for 30 min at RT in the dark, washed in MACS buffer (PBS + 0.5% BSA + 2 mM EDTA, Gibco), and analysed by flow cytometry. MACS-macrophages (7 days) were detached with 6 mg/mL lidocaine (Sigma-Aldrich) and 10 mM EDTA, incubated with Zombie NIR live/dead (Biolegend) for 15 min, blocked with FcR reagent (Miltenyi Biotec) for 20 min at 4°C, and stained with antibodies in MACS buffer for 30 min at 4°C: CD14-BV711 (Biolegend, 301838), CD16-PE/Cy7 (Biolegend, 302016), CD206-APC/Cy7 (Biolegend, 321120), CD163-APC (Biolegend, 333610), CD68-PE/Cy7 (Biolegend, 333816), CD4-PE (Biolegend, 300508), CCR5-APC (Biolegend, 359122), CXCR4-PE/Cy7 (Biolegend, 306514). All samples were analysed on a CytoFLEX Flow Cytometer (Agilent) and processed using FlowJo v10.

Immunofluorescence

Fixed cells were permeabilised and blocked in 1x Perm/Wash buffer (Biolegend) plus 10% FBS. Primary antibodies were incubated overnight at 4°C: OCT-4 (rabbit 1:500, Abcam, ab19857), PAX-6 (mouse 1:200, Abcam, ab78545), BRACHYURY (goat 1:40, Bio-Techne, AF2085), and SOX-17 (goat 1:250, Bio-Techne, AF1924), GFP (rabbit 1:100, Thermo Fisher Scientific, G10362), or PE-p24Gag (1:200, KC57-RD1, Beckman Coulter, 6604667). Appropriate Alexa Fluor-conjugated secondary antibodies were used at 1:500, and nuclei and cytoplasm were counterstained with DAPI and CellMask Deep Red (Invitrogen, A57258). Images were acquired on an Operetta CLS (20x) and analysed using Harmony 4.9 custom pipelines detecting nuclei, cytoplasm, and GFP or Gag per cell (see **Figure S6**), or on a Leica TCS SP8 confocal microscope.

Phagocytosis assay

MACS-macrophages and MDMs (7 days) or undifferentiated iPSCs were incubated with Alexa Fluor 488-conjugated Zymosan A particles (Invitrogen, Z23373) at a 2:1 particle-to-cell ratio for 30 min at 37°C. Non-internalised particles were quenched with 250 µg/mL trypan blue/PBS for 5 min, followed by a PBS wash. As a negative control, MACS-macrophages were pre-treated with 10 µM Cytochalasin D (Tocris, 1233) for 1 h. Cells were analysed by flow cytometry (CytoFLEX, Agilent).

Supplemental References

Adachi, A., Gendelman, H.E., Koenig, S., Folks, T., Willey, R., Rabson, A., and Martin, M.A. (1986). Production of acquired immunodeficiency syndrome-associated retrovirus in human and nonhuman cells transfected with an infectious molecular clone. J Virol *59*, 284–291. https://doi.org/10.1128/jvi.59.2.284-291.1986.

Dull, T., Zufferey, R., Kelly, M., Mandel, R.J., Nguyen, M., Trono, D., and Naldini, L. (1998). A third-generation lentivirus vector with a conditional packaging system. J Virol *72*, 8463–8471. https://doi.org/10.1128/JVI.72.11.8463-8471.1998.

Fouchier, R.A., Meyer, B.E., Simon, J.H., Fischer, U., and Malim, M.H. (1997). HIV-1 infection of non-dividing cells: evidence that the amino-terminal basic region of the viral matrix protein is important for Gag processing but not for post-entry nuclear import. EMBO J *16*, 4531–4539. https://doi.org/10.1093/emboj/16.15.4531.

Rasaiyaah, J., Tan, C.P., Fletcher, A.J., Price, A.J., Blondeau, C., Hilditch, L., Jacques, D.A., Selwood, D.L., James, L.C., Noursadeghi, M., et al. (2013). HIV-1 evades innate immune recognition through specific cofactor recruitment. Nature *503*, 402–405. https://doi.org/10.1038/nature12769.

Zufferey, R., Nagy, D., Mandel, R.J., Naldini, L., and Trono, D. (1997). Multiply attenuated lentiviral vector achieves efficient gene delivery in vivo. Nat Biotechnol *15*, 871–875. https://doi.org/10.1038/nbt0997-871.
